# Molecular Mechanisms and Network Pharmacology Revealing Therapeutic Potential of Acetamidosulfonamides against Parkinsonian Model

**DOI:** 10.1021/acsomega.6c01936

**Published:** 2026-05-21

**Authors:** Waralee Ruankham, Veda Prachayasittikul, Ratchanok Pingaew, Tanawut Tantimongcolwat, Apilak Worachartcheewan, Kanyarat Chompon, Somsak Ruchirawat, Virapong Prachayasittikul, Supaluk Prachayasittikul, Kamonrat Phopin

**Affiliations:** † Department of Clinical Chemistry, Faculty of Medical Technology, 26685 Mahidol University, Bangkok 10700, Thailand; ‡ Center for Research Innovation and Biomedical Informatics, Faculty of Medical Technology, 26685 Mahidol University, Bangkok 10700, Thailand; § Department of Chemistry, Faculty of Science, 37692 Srinakharinwirot University, Bangkok 10110, Thailand; ∥ Department of Community Medical Technology, Faculty of Medical Technology, 26685 Mahidol University, Bangkok 10700, Thailand; ⊥ Laboratory of Medicinal Chemistry, 67969 Chulabhorn Research Institute, Bangkok 10210, Thailand; # Program in Chemical Sciences, Chulabhorn Graduate Institute, Bangkok 10210, Thailand; ∇ Center of Excellence on Environmental Health and Toxicology (EHT), Commission on Higher Education, Ministry of Education, Bangkok 10400, Thailand; ○ Department of Clinical Microbiology and Applied Technology, Faculty of Medical Technology, 26685 Mahidol University, Bangkok 10700, Thailand

## Abstract

Parkinson’s disease (PD) is the second most common age-related motor neurodegenerative disease (ND) that has critically posed a global health burden since the 18th century. There is also no full therapy for the clinical syndrome, nor halts the progression of the disease. This study aimed to investigate multidisciplinary potentials of the acetamidosulfonamides (**1**–**16**) in PD *via* systematic biology-based *in vitro*, *in silico*, and network pharmacology assessments. The biological effects of the synthetic compounds, especially **8**, **13**, **14**, and **16**, provided potent neuroprotective effects against 6-hydroxydopamine (6-OHDA)-induced Parkinsonian SH-SY5Y model through the modulation of antioxidant defenses, antiapoptotic signaling, mitochondrial balance, and the regulation of both acetylcholinesterase (AChE) and sirtuin 1 (SIRT1). Molecular docking confirmed that these synthetic compounds interact favorably with the catalytic active site (CAS) and peripheral anionic site (PAS) of AChE, as well as the active binding pocket of SIRT1. The network pharmacology and target enrichment analysis also revealed a close correlation with APP, MAOA, MAOB, SLC6A3, and DRD1, governing the regulation of neurotransmitters. In summary, this research highlights four acetamidosulfonamides as promising candidates to be further developed as multitarget anti-PD agents for PD prevention and management.

## Graphical abstract

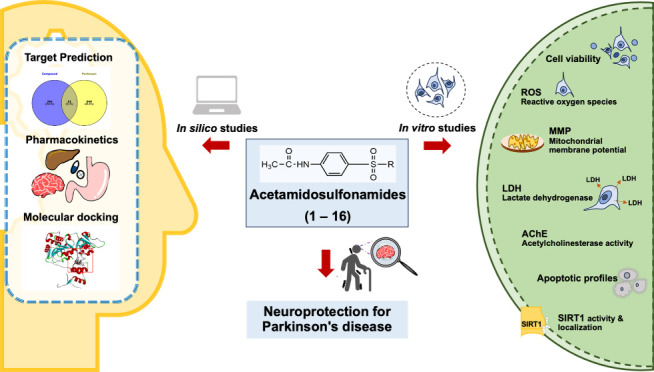

## 1 Introduction

Parkinson’s disease (PD) is one of the second most common neurodegenerative disorders (NDs), following Alzheimer’s disease (AD). It is an irreversibly progressive and chronic neurodegenerative condition with multiple motor (i.e., tremor, rigidity, and bradykinesia) and nonmotor (i.e., dementia, depression, and hyposmia) complications. The disease occurs due to an imbalance between two neurotransmitters, dopamine (DA) and acetylcholine (ACh), in the substantia nigra pars compacta (SNpc). The World Health Organization (WHO) has considered a life-burden impact since the prevalence of PD has been estimated to surpass cancer-related mortality in 2040.^1,2^ Regarding loss of the neurotransmitter DA, current therapies have been focused on DA replacement and ACh modulation to relieve the clinical symptoms but not fully treat the underlying PD pathophysiology.^3–5^ Consequently, there is an urgent need for novel neuroprotective therapeutics that offer high efficacy with minimal adverse effects.

Sulfonamide (−SO_2_NH−), an organosulfur compound, has significantly emerged as a functional pharmacophore in drug discovery and development, providing potential properties to numerous Food and Drug Administration (FDA)-approved drugs targeting multidisciplinary diseases, especially antimicrobial agents.^6,7^ The structure–activity relationship (SAR) analysis revealed that the presence of a diverse range of electron-donating and electron-withdrawing groups is crucial for significant antioxidant activity. To effectively design and discover novel candidates, the modification of sulfonamide core structure was previously demonstrated by our group, particularly thiazole,^8^ phenylene,^9^ and 1,2,3-triazole-based sulfonamides^10^ against 6-hydroxydopamine (6-OHDA)-induced Parkinsonian SH-SY5Y model, as well as anthranilate sulfonamides against hydrogen peroxide-induced AD in SH-SY5Y cells.^11^ Among them, a set of acetamidosulfonamides displayed an attractive SAR model with significant descriptors related to the SOD activity and correlated with antioxidant effects *via* radical scavenging and superoxide dismutase (SOD) activities,^6^ which are noteworthy to be further investigated for their neuroprotective effects.

Herein, the synthetic acetamidosulfonamides (**1**–**16**, Figure 1) were employed to study their neuroprotective roles against 6-OHDA-treated human neuronal SH-SY5Y cells, with a focus on modulation of sirtuin 1 (SIRT1) and ACh under Parkinson-like conditions. In addition to experimental assays, multilevel *in silico* tools (i.e., molecular docking, network pharmacology, target enrichment analysis, and pharmacokinetic prediction) were simultaneously utilized to explore possible underlying mechanisms of these compounds. A combination of *in vitro* and *in silico* approaches might provide fundamental knowledge that paves the way to unravel novel sulfonamide-based drugs by bridging the gap between chemical structure modification and effective therapeutics that might overcome the limitations of current PD preclinical failures.

**Figure 1:**
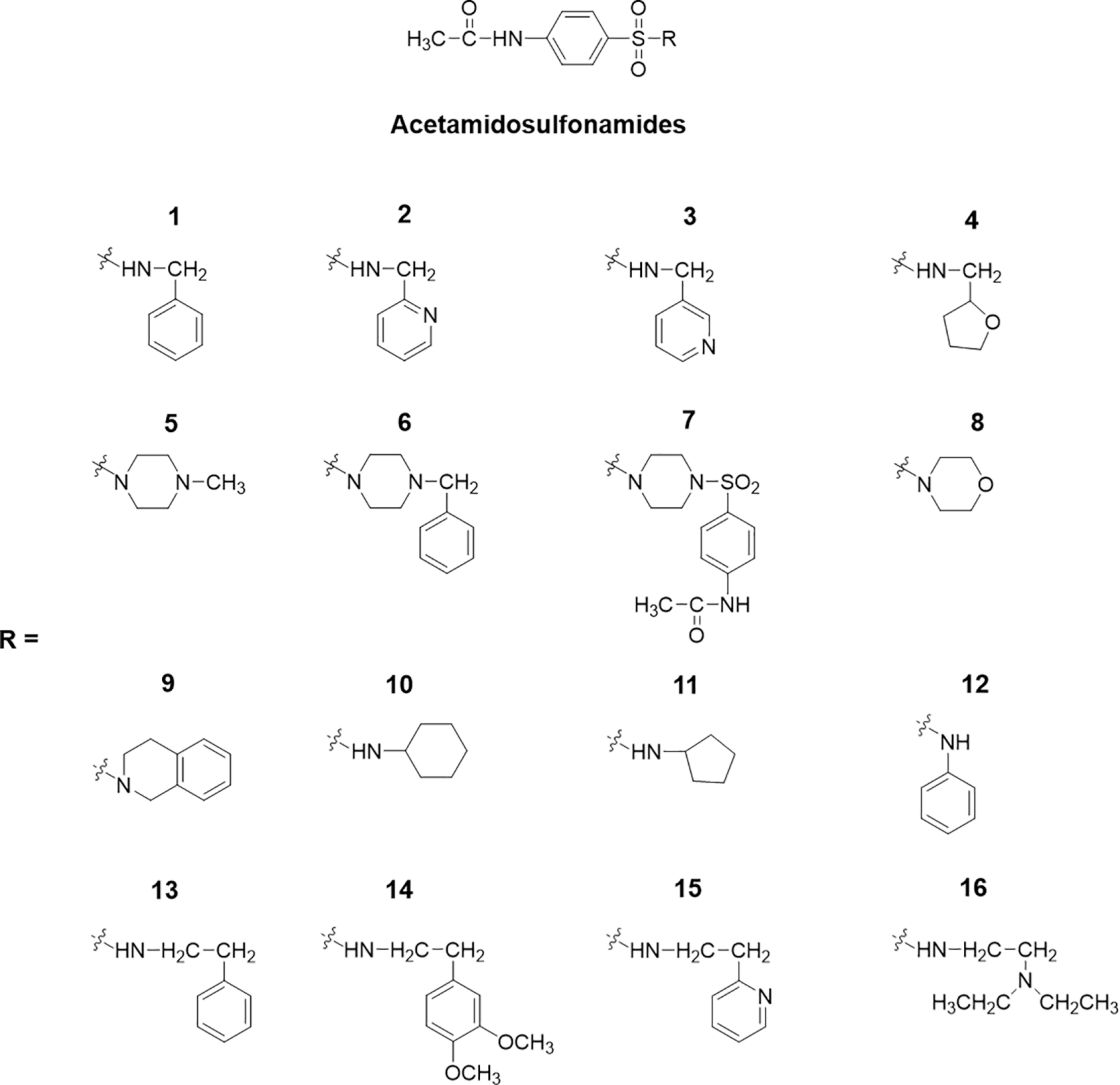
Chemical structures of synthetic acetamidosulfonamides (**1**–**16**).

## 2 Materials and Methods

### 2.1 Chemicals

#### 2.1.1 Acetamidosulfonamide Synthesis

The synthesis of 16 acetamidosulfonamides (**1**–**16**, Figure 1) was based on *N*-sulfonylation as previously reported by our group.^6^ Briefly, 5 mmol of 4-acetamidobenzenesulfonyl chloride in dichloromethane was dropwise added to a stirred mixture of various 5 mmol of amines containing 7 mmol of sodium carbonate in dichloromethane at room temperature. The reaction mixture was extracted and purified by column chromatography on silica gel, followed by structural characterization using HRMS, ^1^H and ^13^C NMR, and IR spectra.

### 2.2 *In Vitro* Studies

#### 2.2.1 Cell Culture and Treatment

Human neuroblastoma SH-SY5Y (CRL-2266) and normal lung fibroblast MRC-5 (CCL-171) cell lines were purchased from the American Type Culture Collection (Manassas, VA, USA). The cells were raised in Dulbecco’s modified Eagle’s medium (DMEM) with the addition of 10% heat-inactivated fetal bovine serum (FBS) and 1% penicillin/streptomycin antibiotic solution (Gibco BRL, MD, USA) at 37 °C in a saturated humidity atmosphere containing 5% CO_2_ and 95% air. The cell culture was replaced with the culture medium every 2–3 days. After reaching 80% confluence, the cells were taken for subsequent experiments by seeding at a density of 1.0 × 10^5^ cells/well in each well plate and incubating overnight.

To investigate the effect of acetamidosulfonamides (**1**–**16**) on 6-OHDA-induced neurotoxicity in the Parkinsonian model, SH-SY5Y cells were pretreated with the compounds (**1**–**16**) for 3 h prior to 100 μM of 6-OHDA (#H4381, Sigma-Aldrich, MO, USA) for an additional 24 h. To further investigate the role of SIRT1 in the neuroprotection of synthetic acetamidosulfonamides, the cells were pretreated with 1 μM of sirtinol (#S7942, Sigma-Aldrich, MO, USA), an SIRT1 inhibitor for 1 h, followed by subjecting them to acetamidosulfonamide pretreatment as mentioned above. A well-known antioxidant, resveratrol (RSV) (# R5010, Sigma-Aldrich, MO, USA) was employed as a positive control, owing to its SIRT1 activator in neuroprotection, as documented in the previous literature.^12^

#### 2.2.2 Cell Morphological Assessment

To indicate the health and function status of the cell, morphological features were quantified as phenotypic metrics (i.e., size, shape, intensity distributions, and subcellular components). The SH-SY5Y cells were treated as described earlier. After the incubation, an inverted light microscope (Olympus Corporation, Tokyo, Japan) at 20× magnification was used to observe the cell morphology. Multiple images were photographed for an individual treatment using a digital camera.

#### 2.2.3 Cell Viability and Cytotoxicity Determinations

To assess the metabolic activity of the cells, capability of mitochondrial dehydrogenase is tracked by reducing 3′-(4,5-dimethylthiazol-2-yl)-2,5-diphenyl tetrazolium bromide (MTT) to its formazan product, which reflects the present number of viable cells.^13^ Briefly, MTT solution (# M6494, Molecular Probes, OR, USA) was added to each well plate and incubated in a dark atmosphere at 37 °C for 3 h. After the incubation, the medium was removed, followed by formazan solubilization with 0.04 N HCl in isopropanol. Cell viability and cytotoxicity were determined using a Multiskan SkyHigh microplate reader (Thermo Fisher Scientific Inc., MA, USA) and a microplate reader (Molecular Devices, CA, USA) at a specific wavelength of 550–570 nm. Doxorubicin and DMSO were used as positive and negative controls, respectively.

#### 2.2.4 Intracellular Reactive Oxygen Species (ROS) Determination

Carboxy-2′,7′-dichlorofluorescein diacetate (DCFDA), a cell-permeable probe, is commonly employed to detect the formation of ROS products in live cells.^14^ After the above-mentioned treatment, the medium was removed, and 10 μM of DCFDA (#D399, Molecular Probes, OR, USA) in phosphate-buffered saline (PBS) was incubated in a dark atmosphere at 37 °C for 1 h. The intracellular ROS generation was determined using an EnSight multimode plate reader (PerkinElmer, MA, USA) at excitation and emission spectra of 495 and 527 nm, respectively.

#### 2.2.5 Mitochondrial Membrane Potential (MMP) Measurement

Rhodamine-123 is a cationic fluorophore used to assess MMP, a key indicator of mitochondrial function.^15^ Briefly, 10 μM of rhodamine-123 (#R8004, Sigma-Aldrich, MO, USA) was added to each well in a dark atmosphere at 37 °C for 30 min. After the incubation, the cells were washed with PBS twice, and MMP levels were detected by an EnSight multimode plate reader at excitation and emission wavelengths of 488 and 525 nm, respectively.

#### 2.2.6 Cell Apoptotic Profile Detection

To detect the apoptotic stages of the cellular membrane integrity, a staining solution of annexin V and 7-aminoactinomycin D (7-AAD) was used as sensitive fluorescent probes discriminating early apoptosis from late apoptosis and necrosis upon the cell membrane.^16^ Following the treatment, both floating and adherent cells were harvested according to the manufacturer’s instructions of Muse annexin V and dead cell kit (#MCH100105) and finally subjected to a Guava Muse cell analyzer (Cytek Biosciences, CA, USA).

#### 2.2.7 Enzymatic Activity Assessments

Lactate dehydrogenase (LDH), a key feature of cells undergoing apoptosis and necrosis, is employed to quantify cellular damage, which is rapidly released into the cell culture medium.^17^ Following the treatment, LDH enzymatic activity was measured using the LDH assay kit (#MAK066, Sigma-Aldrich, MO, USA) based on the conversion of lactate to pyruvate. The colorimetric intensity of the culture medium was measured by a microplate reader at a wavelength of 450 nm.

Acetylcholinesterase (AChE) is a key player in the pathogenesis of NDs.^5^ To monitor neuronal apoptosis against oxidative stress-induced pathological protein aggregation, AChE enzymatic activity was determined using the AChE assay kit (Colorimetric) (#ab138871, Abcam Ltd., Cambridge, UK) by hydrolysis of ACh neurotransmitter. Following the manufacturer’s instructions, the medium was removed and washed with ice-cold PBS, followed by 1× RIPA lysis buffer extraction supplemented with protease inhibitor cocktail (Merck Millipore, Darmstadt, Germany) at 4 °C for 20 min. After centrifugation at 12,000 *g*, 4 °C for 20 min, the supernatant was collected for protein quantification by Bradford protein assay (Bio-Rad Laboratories, Inc., CA, USA). The colorimetric AChE intensity was measured by a microplate reader at a wavelength of 410 nm.

In addition, amelioration of oxidative stress, mitochondrial dysfunction, and apoptosis by SIRT1 activation has contributed to PD neuroprotection.^18^ SIRT1 activity was determined by the SIRT1 assay kit (#CS1040, Sigma-Aldrich, MO, USA). The extracted SIRT1 protein was quantified by a fluorescence microplate reader at an excitation wavelength of 340 nm and an emission wavelength of 445 nm according to the manufacturer.

#### 2.2.8 SIRT1 Immunofluorescent Imaging

To visualize the localization and distribution of SIRT1 protein, immunofluorescence was performed. SH-SY5Y cells were treated as described above. After the incubation, the cells were washed with PBS, followed by an additional incubation of 250 nM MitoTracker dye for mitochondrial labeling (#M7512, Thermo Fisher Scientific Inc., MA, USA) for 30 min at room temperature. After washing with PBS, the cells were fixed in 4% paraformaldehyde for 15 min at room temperature and permeabilized with 1% Triton X-100 in PBS for 10 min at room temperature. Nonspecific targets were blocked with 1% bovine serum albumin (BSA) for 2 h at room temperature. A primary SIRT1 antibody (#8469, Cell Signaling Technology Inc., MA, USA) was added onto the slides at 4 °C overnight, followed by an Alexa Fluor 488 conjugated with antimouse IgG (#4408, Cell Signaling Technology Inc., MA, USA) incubation for 2 h at room temperature. To specify the nucleus localization, DAPI (#4083, Cell Signaling Technology Inc., MA, USA) was used for an additional 10 min of incubation at room temperature. Finally, the slides were mounted and visualized with a microscope BX53 fluorescence with a trinocular head (Olympus Corporation, Tokyo, Japan).

### 2.3 *In Silico* Studies

#### 2.3.1 Physicochemical and Pharmacokinetic Predictions

Drug-likeness and pharmacokinetic profiles (i.e., absorption, distribution, metabolism, excretion, and toxicity; ADMET) of the selected acetamidosulfonamides were computationally evaluated. Briefly, the Simplified Molecular Input Line Entry System (SMILES) formats of the compounds were uploaded as input files for analyses on the three online web tools, including pkCSM (https://biosig.lab.uq.edu.au/pkcsm/), SwissADME (http://www.swissadme.ch), and ProTox-II (http://tox-new.charite.de/protox_II/).

#### 2.3.2 Molecular Docking Simulations

Molecular docking analysis was performed to predict the possible binding modes of the selected acetamidosulfonamides against SIRT1 and AChE targets, using AutoDockTools version 4.2.6.^19^ Crystallographic structures of human SIRT1 (PDB ID: 5BTR) and AChE (PDB ID: 4EY7) complexed with the cocrystallized ligands were retrieved from the Protein Data Bank (PDB). Subsequently, chain A of each protein was prepared by removing the cocrystallized ligands and adding the polar hydrogen atoms. Kollman and Gasteiger charges were added to the protein and investigated compounds, respectively, using AutoDockTools. The AutoGrid was used to generate a grid box of 50 × 50 × 50 and 40 × 40 × 40 centered on the catalytic domain (CD) and N-terminal domain (NTD) of SIRT1 and AChE, respectively, followed by 100 docking runs by the Lamarckian genetic algorithm (LGA). Finally, the least estimated binding energy and positional conformation in the active binding pocket of both proteins were visualized as the most favorable docking pose by Discovery Studio Visualizer 2021 (BIOVIA, Dassault Systèmes, CA, USA). To validate the docking protocol, the cocrystallized ligands, as reference molecules (i.e., RSV and Donepezil), were redocked and calculated for their root-mean-square deviation (RMSD) values. The RMSD between the docked and native conformations of the reference less than 2.0 Å indicates acceptable reliability of the docking protocol.

#### 2.3.3 Network Pharmacology

To construct a reasonable pipeline for network pharmacology, possible protein targets related to PD neuropathology of the 4-acetamidobenzenesulfonyl pharmacophore were extensively explored. Briefly, the selected compounds were uploaded in the SMILES format *via* web-based servers, including SwissTargetPrediction (https://www.swisstargetprediction.ch/), SuperPRED (https://prediction.charite.de/subpages/target_prediction.php), and SEA (https://sea.bkslab.org/) for searching possible protein target-related pharmacophores. Meanwhile, potential protein targets associated with pathological PD were screened in terms of “human” and “Parkinson’s disease” through online databases, including DisGeNET (https://www.disgenet.org/) and GeneCards (https://www.genecards.org). The overlapping protein association of interested compounds and protein-associated diseases was visualized by online Venny 2.1.0 (https://bioinfogp.cnb.csic.es/tools/venny/) and further analyzed using STRING database version 12.0 (https://string-db.org/). Finally, compound–target disease (CTD) and protein–protein interaction (PPI) networks were created using open-source software Cytoscape 3.10.1 (https://cytoscape.org/). An illustration of the top 20 pathways was performed using ShinyGO 0.82 (http://bioinformatics.sdstate.edu/go/).

### 2.4 Statistical Analysis

One-way analysis of variance (ANOVA), followed by a Tukey–Kramer post hoc test, was performed by GraphPad Prism 6 scientific software (GraphPad Software Inc., CA, USA). All data were representative of three independent experiments and expressed as mean ± standard error of the mean (SEM). Probability (*p* < 0.05) is considered statistically significant.

## 3 Results

### 3.1 Acetamidosulfonamides Inhibit 6-OHDA-Induced Cytotoxicity in MRC-5 and SH-SY5Y Cells

A series of 16 acetamidosulfonamides (**1**–**16**) has a common 4-acetamidobenzenesulfonyl pharmacophore bearing diverse amino substituents (R), including (A) methylamino (**1**–**4**), (B) cyclic amino (**5**–**9**), (C) ring-substituted amino (**10**–**12**), and (D) ethylamino (**13**–**16**) moieties. To investigate the cytotoxic effect of these compounds in human MRC-5 and SH-SY5Y cells, the viability of cells was assessed using the colorimetric MTT assay. The IC_50_ value was determined as the concentration of the tested derivative required for 50% inhibitory effect. The compound exhibiting an IC_50_ value over 50 μg/mL was considered to have no cytotoxic effect. All derivatives (**1**–**16**) displayed non-cytoxic to the normal lung MRC-5 cells when compared to the reference doxorubicin drug (Table S1). As shown in Figure 2, the SH-SY5Y cells were pretreated with acetamidosulfonamides (**1**–**16**) at different concentrations ranging from 0.1 to 100 μM. These compounds did not significantly influence the cell viability compared to the untreated cells.

**Figure 2:**
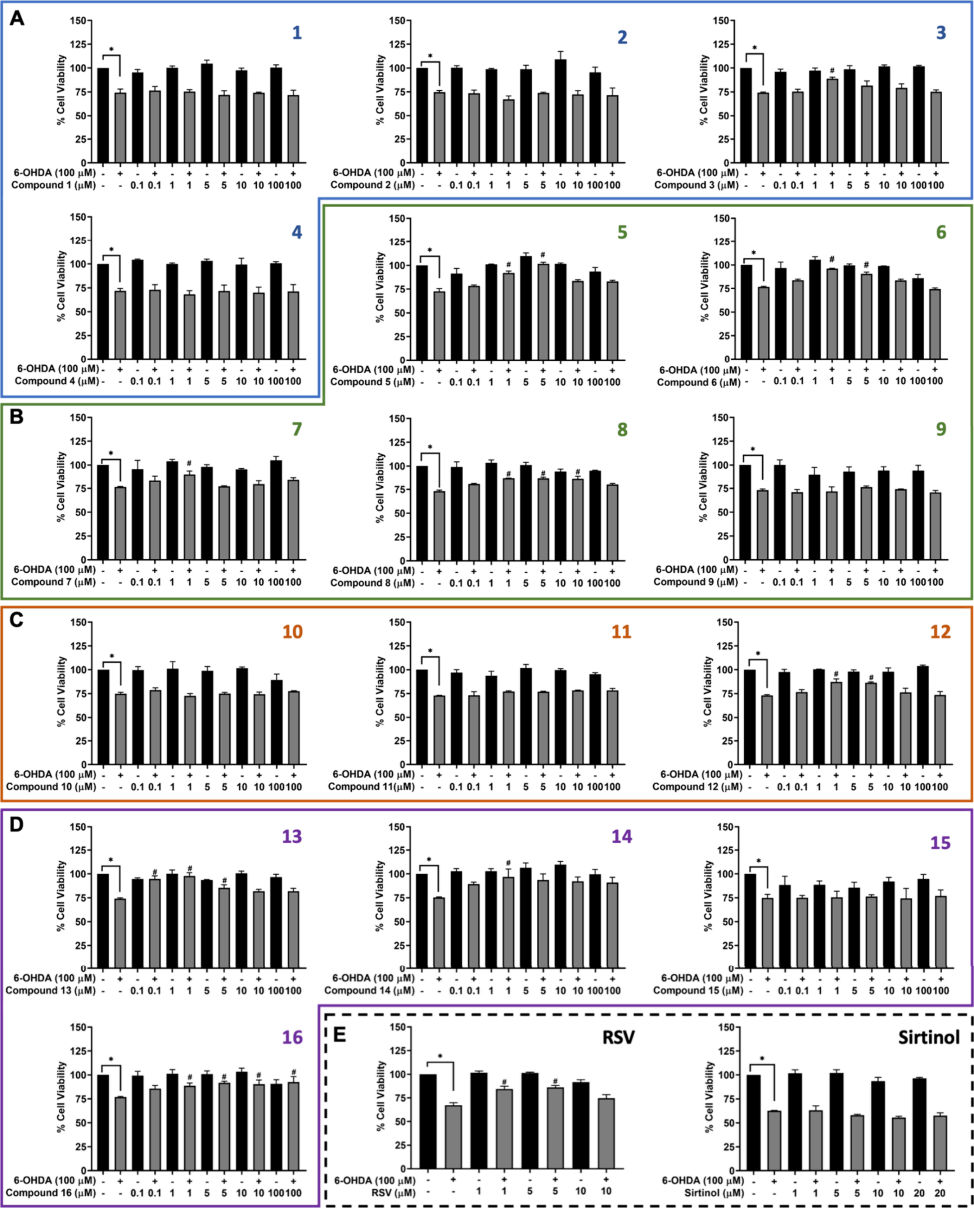
Cell viability of 16 acetamidosulfonamides (**1**–**16**) in 6-OHDA-induced SH-SY5Y cells by MTT assay. (A) methylamino (**1**–**4**), (B) cyclic amino (**5**–**9**), (C) ring-substituted amino (**10**–**12**), (D) ethylamino (**13**–**16**), and (E) references (RSV and sirtinol). The data are presented as the mean ± SEM. *p < 0.05 vs control and ^#^ *p* < 0.05 vs 6-OHDA.

6-OHDA, a common neurotoxin that has been widely employed to mimic the PD model, was chosen in this study. According to a sublethal window in *in vitro* neuroprotective studies,^8,9^ 100 μM 6-OHDA-treated SH-SY5Y cells significantly decreased the cell viability to approximately 74.31 ± 1.37% compared to the untreated cells. However, this effect was abolished by the acetamidosulfonamide pretreatment, especially cyclic amino (Figure 2B), including 1 μM of compounds **5**, **6**, **7**, and **8** (91.95 ± 2.04, 96.10 ± 0.45, 90.09 ± 3.66, and 87.30 ± 0.19%, respectively), 5 μM of compounds **5**, **6**, and **8** (101.6 ± 1.68, 90.60 ± 1.70, and 87.22 ± 1.19%), and 10 μM of compound **8** (86.64 ± 2.67%), and ethylamino (Figure 2D) groups, including 0.1 μM of compound **13** (94.73 ± 3.25%), 1 μM of compounds **13** and **14** (97.72 ± 3.59 and 96.82 ± 8.39%), and 5 μM of compound **13** (85.43 ± 3.17%). Particularly, compound **16** showed the most effectiveness at concentrations of 1, 5, 10, and 100 μM (88.59 ± 1.73, 91.74 ± 0.96, 90.39 ± 2.49, and 92.65 ± 3.24%, respectively). On the other hand, there was no effect observed by compounds **9** and **15**. Groups A and C of methylamino (Figure 2A) and ring-substituted amino (Figure 2C) also showed no recovery of viability against the 6-OHDA exposure, except for 1 μM of compound **3** (88.81 ± 1.60%), and 1 and 5 μM of compound **12** (87.30 ± 0.19 and 87.22 ± 1.19%).

In addition, RSV, a well-known phenolic antioxidant and SIRT1 activator, has been shown to prevent the loss of dopaminergic neurons in both *in vitro* and *in vivo* Parkinsonian models.^20^ As shown in Figure 2E, the RSV treatment at concentrations of 1 and 5 μM remarkably improved the viability of the neuronal cells (84.39 ± 2.96 and 86.01 ± 2.20%) compared to the 6-OHDA group, similarly to the above-mentioned acetamidosulfonamide moieties. The varying concentrations from 1 to 20 μM of sirtinol did not influence the cell viability. Therefore, the selected acetamidosulfonamides (**3**, **5**, **6**, **7**, **8**, **12**, **13**, **14**, and **16**) at a concentration of 1 μM were selected for subsequent experiments.

### 3.2 Acetamidosulfonamides Exhibit Neuroprotection against 6-OHDA-Induced Neuronal Death in SH-SY5Y Cells

Mitochondrial function, a key indicator of cell health, can be assessed by monitoring the changes in ROS formation^15^ and MMP.^17^ To extend the *in vitro* cell-free antioxidant assays of acetamidosulfonamides by radical scavenging and SOD activities,^6^ the intracellular ROS production and MMP of acetamidosulfonamides-pretreated cells in 6-OHDA-induced oxidative stress were examined by fluorescent DCFDA (Figure 3A) and rhodamine-123 (Figure 3B) stainings. The results showed that 100 μM 6-OHDA dramatically increased the ROS products to 138.0 ± 7.47% but decreased MMP to 77.49 ± 0.80% compared to the untreated group, indicating the induction of oxidative stress. On the other hand, pretreatment with the selected acetamidosulfonamides (**3**, **5**, **6**, **7**, **8**, **12**, **13**, **14**, and **16**) at a concentration of 1 μM showed no significant effects against the ROS production and MMP within the cells. All the investigated compounds also diminished the negative effects against 6-OHDA-induced oxidative damage (106.41 ± 1.25, 104.64 ± 3.33, 101.81 ± 2.05, 106.03 ± 2.21, 99.23 ± 3.16, 102.88 ± 3.35, 97.33 ± 5.69, 103.99 ± 4.45, and 105.96 ± 5.89%, respectively) in a similar manner to the well-known antioxidant RSV (ROS 110.26 ± 2.83% and MMP 95.29 ± 2.21%). However, the compounds **6**, **7**, and **12** ineffectively improved the MMP activity (88.55 ± 0.50, 88.75 ± 2.96, and 87.15 ± 3.12%, respectively). These indicated antioxidant properties of acetamidosulfonamides (**3**, **5**, **8**, **13**, **14**, and **16**) in balancing the ROS production and antioxidant defense, leading to proper mitochondrial function (96.14 ± 0.91, 92.54 ± 1.93, 95.15 ± 1.59, 95.19 ± 3.27, 93.94 ± 0.48, and 92.41 ± 1.95%, respectively).

**Figure 3:**
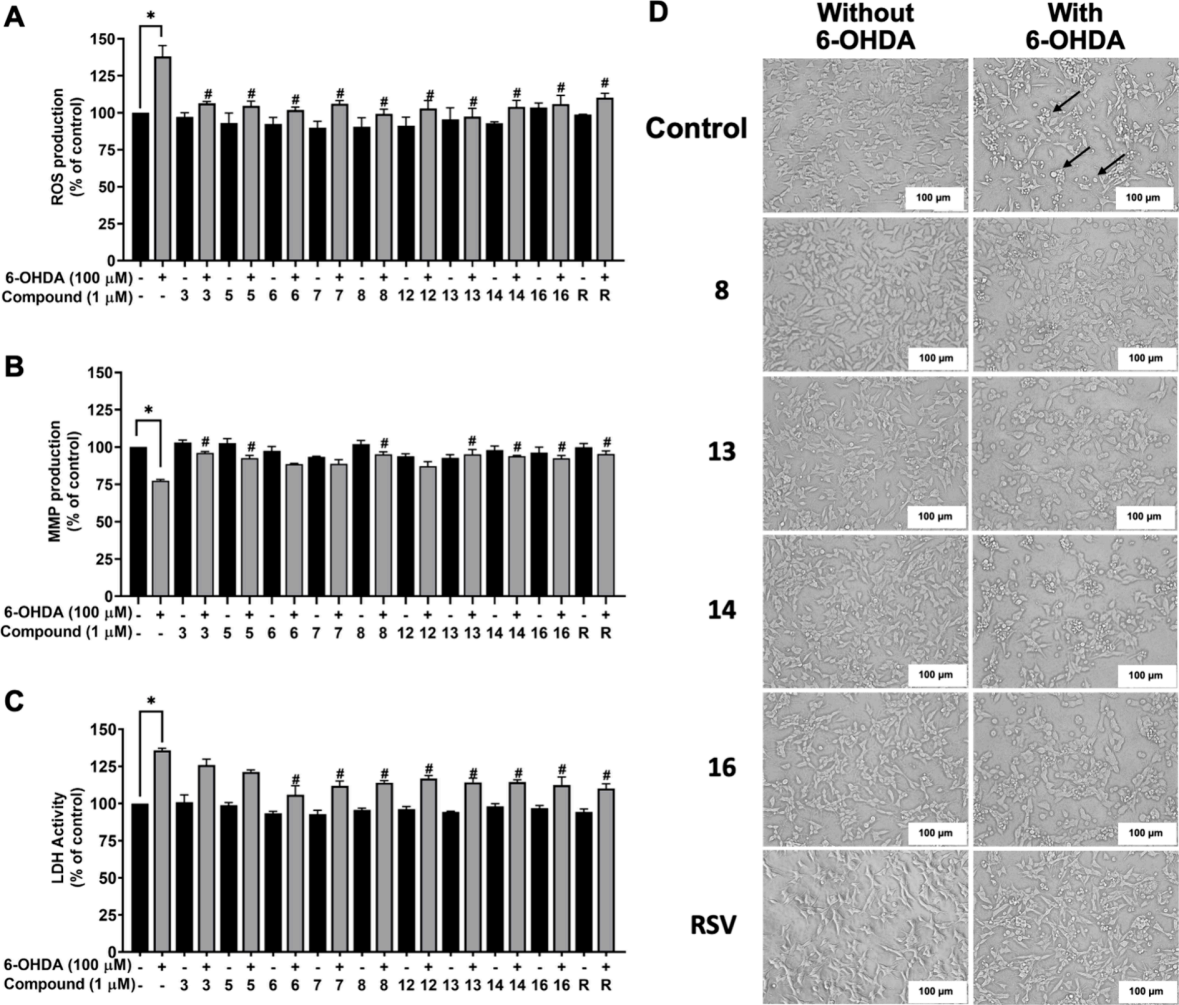
Neuroprotective properties of acetamidosulfonamides in 6-OHDA-treated SH-SY5Y cells. (A) Intracellular ROS production by DCFDA fluorescence staining, (B) MMP activity by rhodamine-123 staining, (C) LDH leakage by LDH enzymatic assay, and (D) cellular morphology by microscopy at 20× magnification and scale bar of 100 μm. The arrow depicts the apoptotic-like morphology. The data are presented as the mean ± SEM. *p < 0.05 vs control and ^#^ *p* < 0.05 vs 6-OHDA.

An indicator of cell damage, like LDH enzyme, was also evaluated. As shown in Figure 3C, 100 μM 6-OHDA remarkably induced the leakage of LDH up to 135.81 ± 1.39%, indicating damage to the neuronal cell membrane. All acetamidosulfonamides (**3**, **5**, **6**, **7**, **8**, **12**, **13**, **14**, and **16**) did not affect the release of LDH. A significant decrease in LDH changes was found in acetamidosulfonamides (**6**, **7**, **8**, **12**, **13**, **14**, and **16**)-pretreated SH-SY5Y cells (105.88 ± 6.06, 111.88 ± 3.27, 114.10 ± 1.38, 116.88 ± 2.06, 114.25 ± 2.92, 114.47 ± 1.57, and 112.39 ± 5.53%, respectively) compared to the 6-OHDA condition, while compounds **3** and **5** (125.88 ± 4.01 and 121.40 ± 1.26%) were not able to diminish the overproduction of LDH. These were in line with the potential effect of RSV (110.10 ± 3.20%). Therefore, the acetamidosulfonamides providing antioxidant (**8**, **13**, **14**, and **16**) were selected for subsequent investigation of the underlying PD mechanism.

Morphological changes of neuronal cells were further observed under the 20× magnifying light microscope (Figure 3D). The appearance of 100 μM 6-OHDA-treated SH-SY5Y cells showed the changes of cellular morphology with contracted and fell off spherically, blebbing, and cell shrinkage compared to the control. These features can be indicative of the cells undergoing apoptosis. As expected, the above-mentioned cell morphology was not observed in acetamidosulfonamides (**8**, **13**, **14**, and **16**)-pretreated SH-SY5Y cells, and only slight morphological changes were observed after exposure to 6-OHDA.

Subsequently, the effect of acetamidosulfonamides on apoptosis under 6-OHDA treatment was investigated by annexin V and 7-AAD staining. Flow cytometric scattergrams of apoptotic profiles are represented in Figure 4A. In each quadrant, the lower left population shows living cells, which are negative for both stainings of annexin V and 7-AAD. The lower right population shows early apoptotic cells, which are positive only for annexin V. The upper right population shows late apoptotic cells, which are positive for both stainings of annexin V and 7-AAD. Lastly, the upper left population shows death or necrotic cells, which are positive only for 7-AAD staining. Results revealed that the SH-SY5Y cells treated with 100 μM 6-OHDA had a significantly reduced cell survival rate to 54.63%, specifically 29.13% of cells entered the early apoptosis, 15.34% of the late apoptosis, and 0.9% of death when compared to the untreated group. Pretreatment of 1 μM acetamidosulfonamides (**8**, **13**, **14**, and **16**) considerably inhibited the total apoptotic percentage from early (18.00, 17.07, 18.13, and 22.73%, respectively) to late apoptotic (5.57, 4.16, 4.03, and 4.30%, respectively) phases, in comparison to those of the 6-OHDA-induced cell death. A slight effectiveness of the selected compounds was found when compared to the total apoptosis of RSV (early 13.94% and late 3.53% stages).

**Figure 4:**
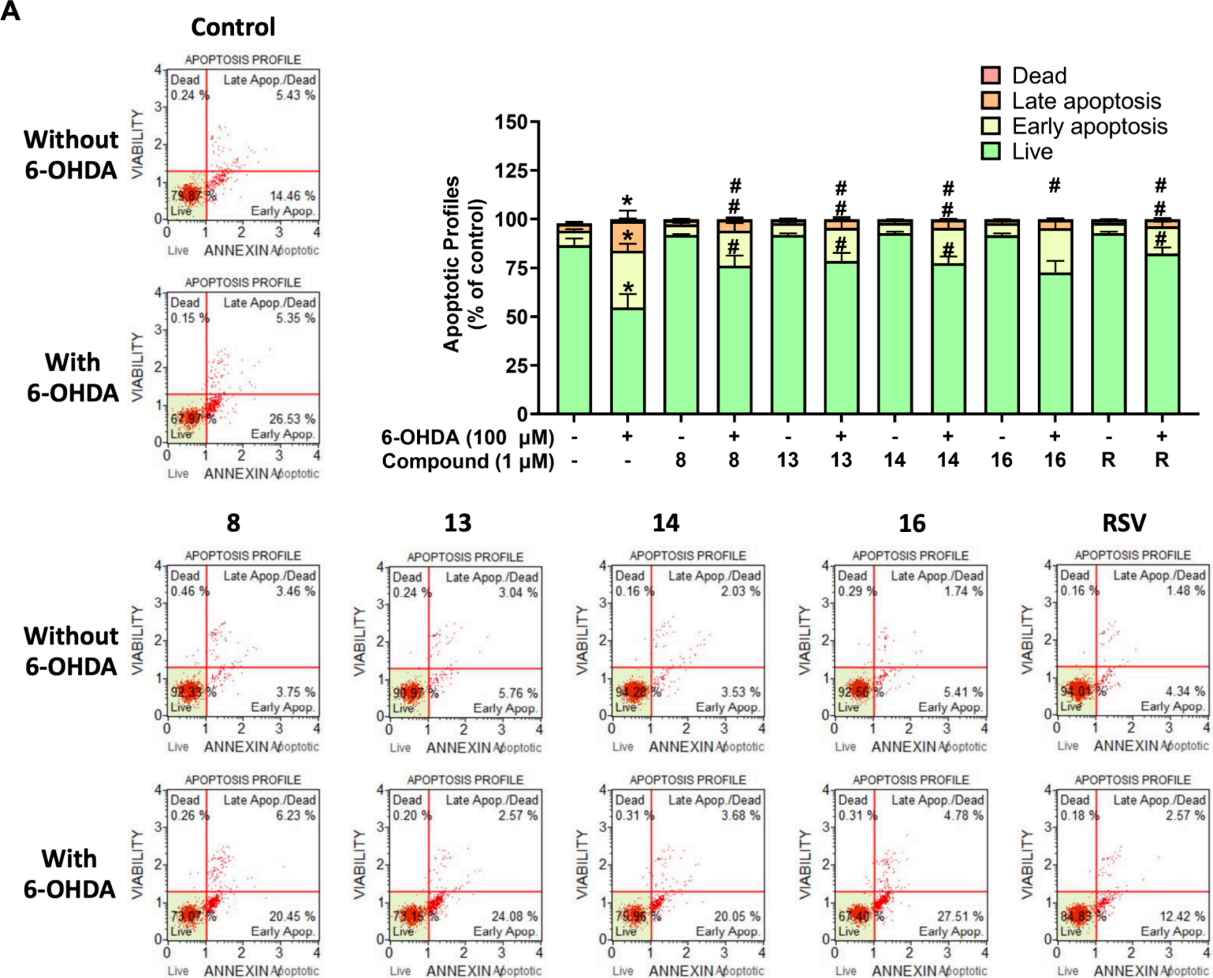

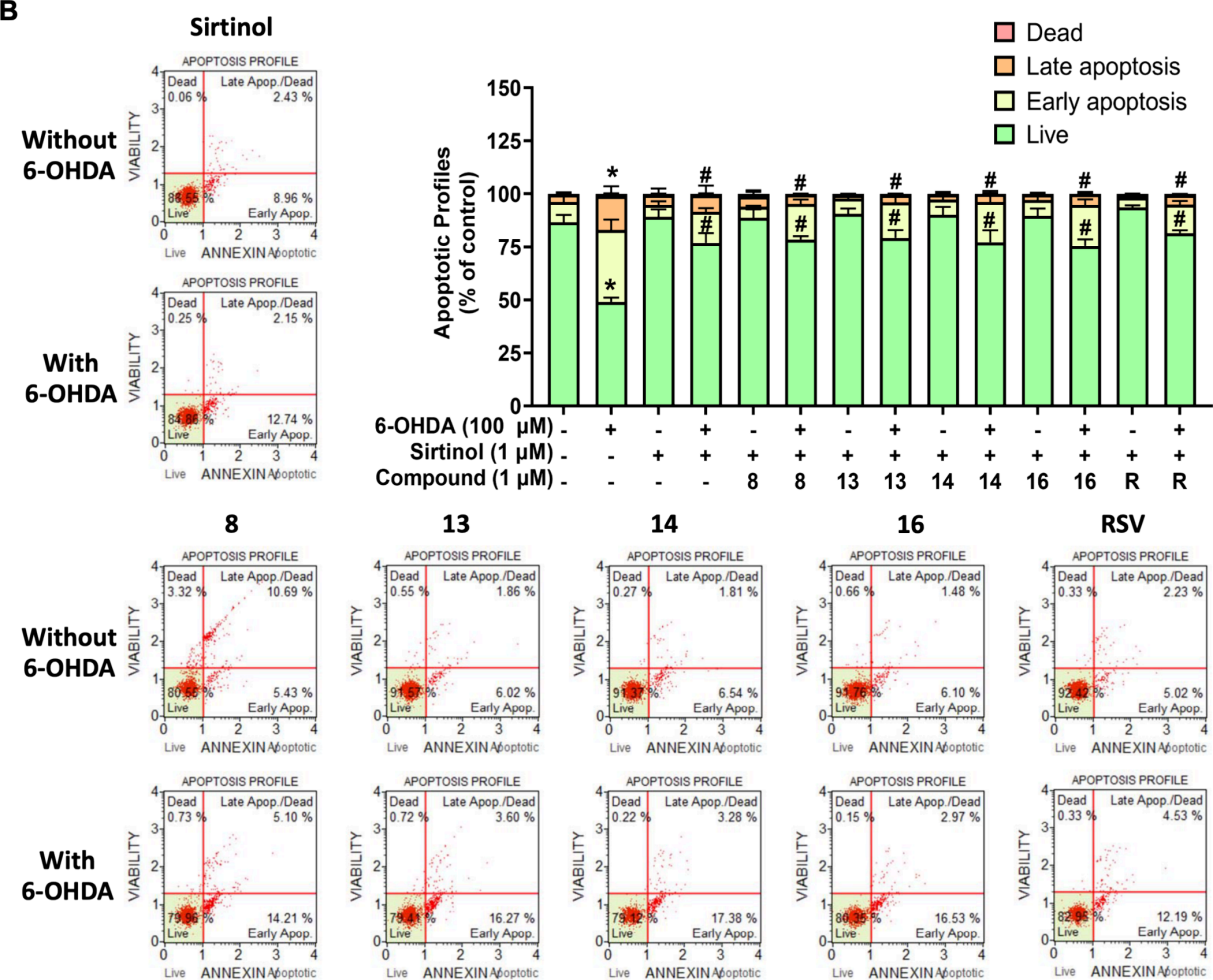
Apoptotic profiles of acetamidosulfonamides against 6-OHDA-induced SH-SY5Y cells. Flow cytometric scattergrams and histograms (A) without and (B) with sirtinol. The data are presented as the mean ± SEM. *p < 0.05 vs control and ^#^ *p* < 0.05 vs 6-OHDA.

The effect of a sirtinol on apoptosis was also examined (Figure 4B). Sirtinol treatment alone had no irritation compared to the control. While the SH-SY5Y treated with 1 μM sirtinol for 1 h prior exposure to 100 μM 6-OHDA significantly increased cell survival rate to 76.83%, and the cells entered early and late apoptotic phases of 14.7 and 7.87%, respectively, compared to the 6-OHDA-induced neuronal death. The necrotic cells were observed at approximately 0.6%. Importantly, copretreatment of sirtinol and acetamidosulfonamides (**8**, **13**, **14**, and **16**) showed no effect on the apoptotic profiles compared to the untreated cells. However, the acetamidosulfonamide-elicited effect on apoptosis was attenuated by 6-OHDA exposure with a slight decrease of the early (16.83, 16.74, 19.00, and 19.27%, respectively) and late apoptosis (4.37, 3.6, 3.8, and 5.07%, respectively), similar to the RSV efficiency (early 13.57% and late 4.6% stages). These results suggested that the compounds **8**, **13**, **14**, and **16** exhibit neuroprotective effects against 6-OHDA-induced oxidative damage through improving cell viability, balancing antioxidants, mitigating MMP, decreasing LDH leakage, and ultimately reducing apoptosis.

### 3.3 Acetamidosulfonamides Regulate AChE and SIRT1-Related Neuroprotection

AChE plays an important role in NDs, influencing the response to inflammation, apoptosis, oxidative stress, and the aggregation of pathogenic proteins. Impaired AChE activity has been observed in patients living with dementia. The decrease in enzyme activity in PD is related to the peripheral cholinergic degeneration, which is also independent of motor function and severity of the disease.^4^ Herein, the alteration in AChE activity was observed to provide valuable insights into the association between the effect of acetamidosulfonamides and cholinergic dysfunction of PD driven by 100 μM 6-OHDA-treated neuronal cells (Figure 5A). The results showed that a remarkable decrease in AChE activity was found (57.09 ± 5.52%) in the 6-OHDA exposure compared to the control. However, pretreatment with acetamidosulfonamides (**8**, **13**, **14**, and **16**) did not change the AChE activity and significantly helped restore the impairment of AChE against 6-OHDA-treated neuronal cells (77.07 ± 1.82, 88.11 ± 5.75, 79.18 ± 2.36, and 77.18 ± 5.20%, respectively), which was in a similar manner to that of the RSV (81.40 ± 4.36%).^21^ These indicated that the selected acetamidosulfonamides have the ability to balance the levels and function of AChE in the PD condition.

**Figure 5:**
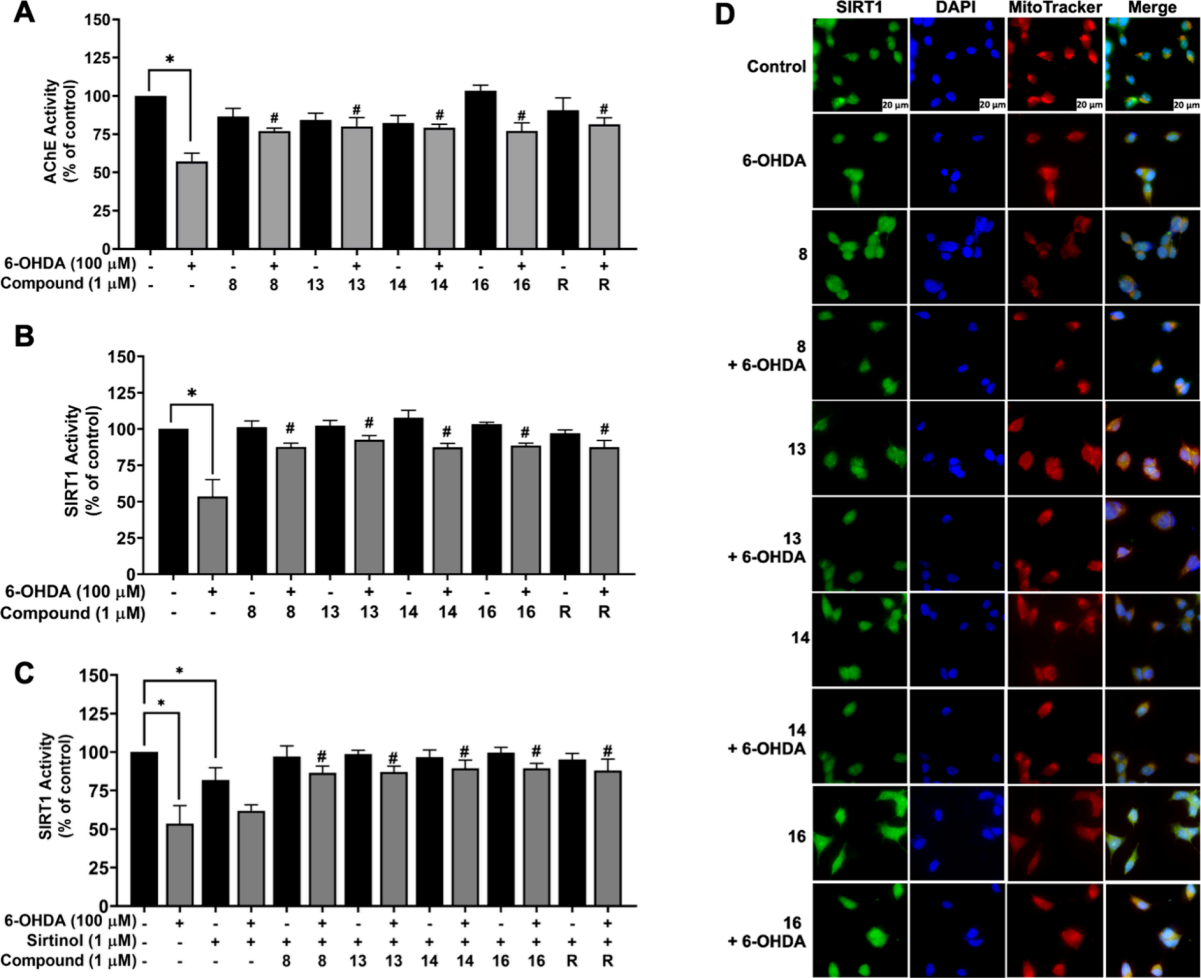
Neuroprotective effects of acetamidosulfonamides against 6-OHDA-treated SH-SY5Y cells. Activities of (A) AChE, (B) SIRT1 without and (C) with sirtinol inhibitor, as well as (D) SIRT1 localization at 60× magnification and scale bar of 20 μm. The data are presented as the mean ± SEM. *p < 0.05 vs control and ^#^ *p* < 0.05 vs 6-OHDA.

Currently, the therapeutic potential of SIRTs family has been extensively explored to promote lifespan extension and exert protection against age-associated NDs, including PD. The family of SIRTs (SIRT1–7) enzymes belongs to a nicotinamide adenosine dinucleotide (NAD)-dependent deacetylase and plays a critical role in modulating cellular functions, with an imbalance of SIRTs activity and NAD levels observed in aging, particularly SIRT1.^22,23^ The synaptic formation and activity-activated by SIRT1 can also mitigate the progression of various NDs, as well as neuroinflammation^24^ and aggregation of alpha-synuclein (α-syn), a protein implicated in PD.^18,25^ After treatment with 100 μM 6-OHDA (Figure 5B), the reduction of SIRT1 activity was significantly observed to 53.46 ± 11.73% compared to the untreated cells, while pretreatment with 1 μM acetamidosulfonamides (**8**, **13**, **14**, and **16**) or RSV did not affect the SIRT1 levels and also mitigated the toxicity of 6-OHDA by the activation of SIRT1 (83.95 ± 1.89, 89.48 ± 4.77, 83.43 ± 2.25, 84.42 ± 2.55, and 85.31 ± 4.19%, respectively).

To confirm the extensive role of SIRT1 function in acetamidosulfonamides-pretreated neuronal cells, sirtinol was employed. As mentioned earlier, the SIRT1 inhibition by sirtinol was confirmed due to the decline of SIRT1 to 81.81 ± 7.89% and even lower to 61.84 ± 3.85% after the 6-OHDA exposure compared to the control (Figure 5C). As expected, the exposure of 1 μM sirtinol, followed by the treatment of four compounds **8**, **13**, **14**, and **16** significantly exhibited the protective effects of SIRT1 activation up to 86.37 ± 4.51, 86.99 ± 3.82, 86.38 ± 5.35, and 89.34 ± 3.28%, respectively similar to the SIRT1 activator, RSV (88.06 ± 7.35%) compared to the 6-OHDA alone. Furthermore, the results were concurrent with the localization of nuclear SIRT1 protein by immunofluorescence staining. Representative immunofluorescence images of SIRT1 (green), DAPI-stained nuclei (blue), and MitoTracker-stained mitochondria (red) were counterstained with the merged panel (Figure 5D). The result revealed that acetamidosulfonamides maintained 6-OHDA-caused SIRT1 reduction in the nucleus of dopaminergic cells and reversed the fluorescent signal of sirtinol-induced SIRT1 inhibition and RSV-induced SIRT1 activation (Figure S1). Thus, acetamidosulfonamides-activated SIRT1 might play a crucial role in the mitigation of PD neuropathology through the downstream signaling pathways of oxidative stress, mitochondrial dysfunction, and apoptosis.

### 3.4 Acetamidosulfonamides Bind to the Active Binding Site of AChE and SIRT1 Proteins

Molecular docking was performed to reveal binding behaviors of the compounds against the target protein SIRT1 (PDB ID: 5BTR).^26^ Redocking of cocrystallized RSV provided an acceptable RMSD value (RMSD = 0.730 Å), indicating the reliability of the docking protocol (Figure 6A). The validated simulation was further used to investigate binding modes of the selected compounds (**8**, **13**, **14**, and **16**). It was revealed that all investigated compounds could occupy within the same active binding site of the RSV, suggesting that they possibly acted as SIRT1 activators. All compounds displayed better binding affinity with SIRT1, as indicated by their lower calculated free binding energy values (**8**, **13**, **14**, and **16**: −8.07, −9.39, −9.58, and −7.77 kcal/mol, respectively) when compared to that of RSV (−7.53 kcal/mol). The studied compounds displayed some common interacting residues with those of RSV, including Leu202, Thr209, Pro211, Ile223, and Asn226. It was shown that the binding of RSV with SIRT1 is governed by multiple interactions, in which the two-terminal benzene rings were noted as an essential feature required for the formation of π-alkyl (with Leu202, Ile223, Pro211, and Arg446) and van der Waals interaction (with Asn226), whereas its substituted hydroxyl groups played a role in conventional bonding interaction (with Thr209 and Glu230) (Figure S2A). Among others, compounds **13** and **14** provided the most preferable affinity, which was suggested to be due to the presence of the substituted benzene ring (R) in their molecules that could mimic the binding of the two-terminal benzene rings of the RSV. Additionally, their sulfonyl oxygen atoms played a key role in the formation of conventional bonds with Asn226. The superior binding affinity of compound **14** beyond **13** could be due to the presence of additional substitutions of two OCH_3_ groups on the terminal benzene (R) that facilitate the multiple formations of π- and/or alkyl interactions, as well as a carbon–hydrogen bond interaction with Thr209 to mimic a benzene and its substituted hydroxyl group of the RSV.

**Figure 6:**
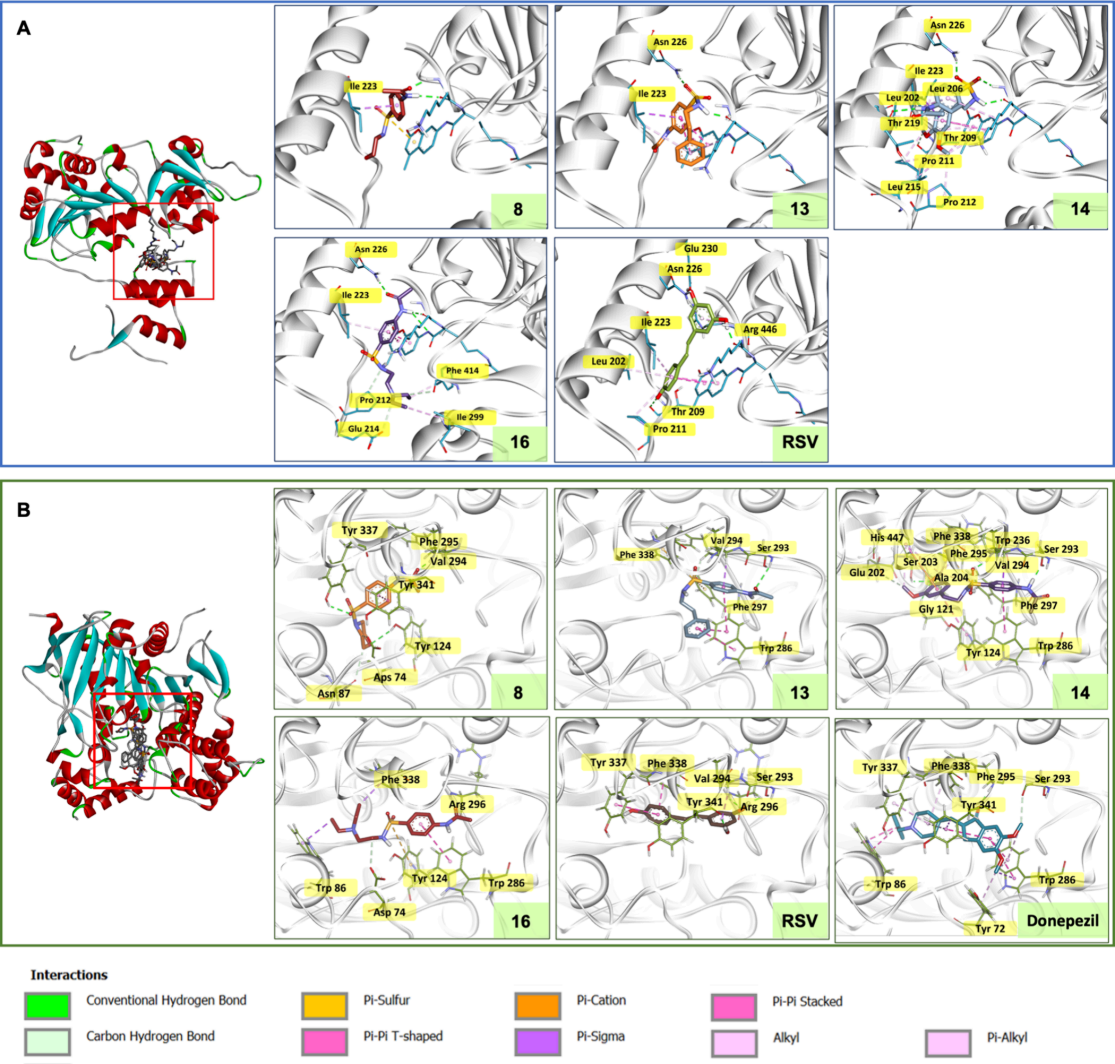
Molecular docking of acetamidosulfonamides in the active binding site of (A) SIRT1 and (B) AChE.

The selected compounds were also investigated for their binding modes against AChE in complex with the anti-dementia drug, Donepezil (PDB ID: 4EY7).^27^ Redocking of cocrystallized donepezil provided an RMSD value of 0.7 Å, suggesting an acceptable accuracy of the simulation. The tested compounds (**8**, **13**, **14**, and **16**: −7.73, −8.52, −9.18, and −7.80 kcal/mol, respectively) had comparable binding affinities to the reference drug (−10.48 kcal/mol), as well as the RSV (−7.3 kcal/mol). AChE crystallography reveals a narrow dumbbell-shaped channel of the ligand-binding pocket, containing catalytic anionic site (CAS) and peripheral anionic site (PAS) domains.^28^ Molecular docking studies demonstrated that π-based interactions play the key roles in the binding modes of the compounds against the AChE (i.e., **8** with Tyr341, **13** with Trp286 and Val294, **14** with Tyr124, Ala204, Trp236, Trp286, and Val294, as well as **16** with Trp86, Trp286, and Phe338). Conventional hydrogen and carbon–hydrogen also played roles in the binding with AChE, such as **8 (**Asp74, Asn87, Tyr124, Val294, Phe295, and Tyr337), **13** (Ser293), **14** (Gly121, Glu202, Ser203, Ser293, and Phe295), and **16** (Asp74 and Arg296). This was predominantly in line with both π-interactions (Trp86, Trp286, Tyr337, Phe338, and Tyr341) and hydrogen bonds (Ser293 and Phe295) of the reference Donepezil. Additional π–sulfur interactions were observed between three compounds with the lowest binding free energy values (i.e., **14** > **13** > **16**: binding energy values −9.18 > −8.52 > −7.80 kcal/mol, respectively). These π–sulfur interactions were found between the sulfonyl group of compounds **14** and **13** with Phe297 and Phe338 in the Mid-Gorge between the PAS and CAS pockets, as well as that of compound **16** with Tyr124 in the PAS pocket, a binding site for the enzymatic inhibitor. This suggested that the presence of sulfonamide moiety is essential for facilitating the dual-site binding of these acetamidosulfonamides. Furthermore, the most preferable binding behavior of compound **14** could also be facilitated by an additional π–cation interaction formed between its terminal benzene ring and His447 residue in the catalytic triad for substrate and inhibitor binding sites (Figure S2B). In overview, the binding affinity of these four compounds showed a similar trend against two targets, in which compound **14** demonstrated the best affinity, followed by compounds **13**, **16**, and **8**, respectively. Notably, all compounds showed superior binding affinity than the known SIRT1 activator, RSV, against both targets, suggesting their potentials to activate the SIRT1 and AChE proteins, as well as regulate the downstream pathway-related PD.

### 3.5 Acetamidosulfonamides Interact with PD-Related Proteins

Expanding exploration of the possible target proteins among acetamidosulfonamides and PD is crucial for identifying downstream key signatures associated with neuroprotection. As illustrated in Figure 7A, the Venn diagram represents the overall key proteins of the investigated compounds (**8**, **13**, **14**, and **16**) and PD, showing that 345 targets were unique to the online SwissTargetPrediction-, SEA-, and SuperPRED-based compounds, and 700 were exclusive to the PD targets from GeneCards and DisGeNET databases. Utilizing an integrative Venn diagram approach, 51 overlapping targets were identified and regarded as potential targets in acetamidosulfonamides-induced neuroprotection in humans. The core relationship between key active PD and key targets of the compounds **8**, **13**, **14**, and **16** was identified as the CTD network with the anticipation of 27, 37, 33, and 29 targets, respectively (Figure 7B). The PPI network was constructed using the string platform with the highest confidence level of 0.9 and further analyzed the centralized parameters based on degree (DC), betweenness (BC), closeness (CC), and network of the nodes using Cytoscape (Table 1). On the basis of node degree, the top 18 key nodes were identified and are presented in Figure 7C. The top five nodes in terms of degree values are APP, MAOA, MAOB, SLC6A3, and DRD1, which uncovered an association with the primary cellular functions (i.e., cell cycle, DNA damage, cell survival, metabolism, apoptosis, and inflammation) to the regulation of neurotransmitters. Concurrently, the SIRT1 and a hydrolytic enzymatic family of AChE, butyrylcholinesterase (BChE), revealed a primary top 18 association with the PD signaling pathways, which confirmed the active functions of the preliminary enzymatic activities. These 18 key targets underwent a comprehensive functional enrichment using the ShinyGO pathway (Figure 7D) and GO enrichment analyses (Figure 7E), which revealed the top 20 most enriched pathways with a particular emphasis on NDs, especially cognition disorders, sporadic PD, drug abuse, psychological stress, and memory impairment (Table 2), clarifying the involvement in similar biological functions and neurobiological signaling pathways (Figure S3).

**Figure 7:**
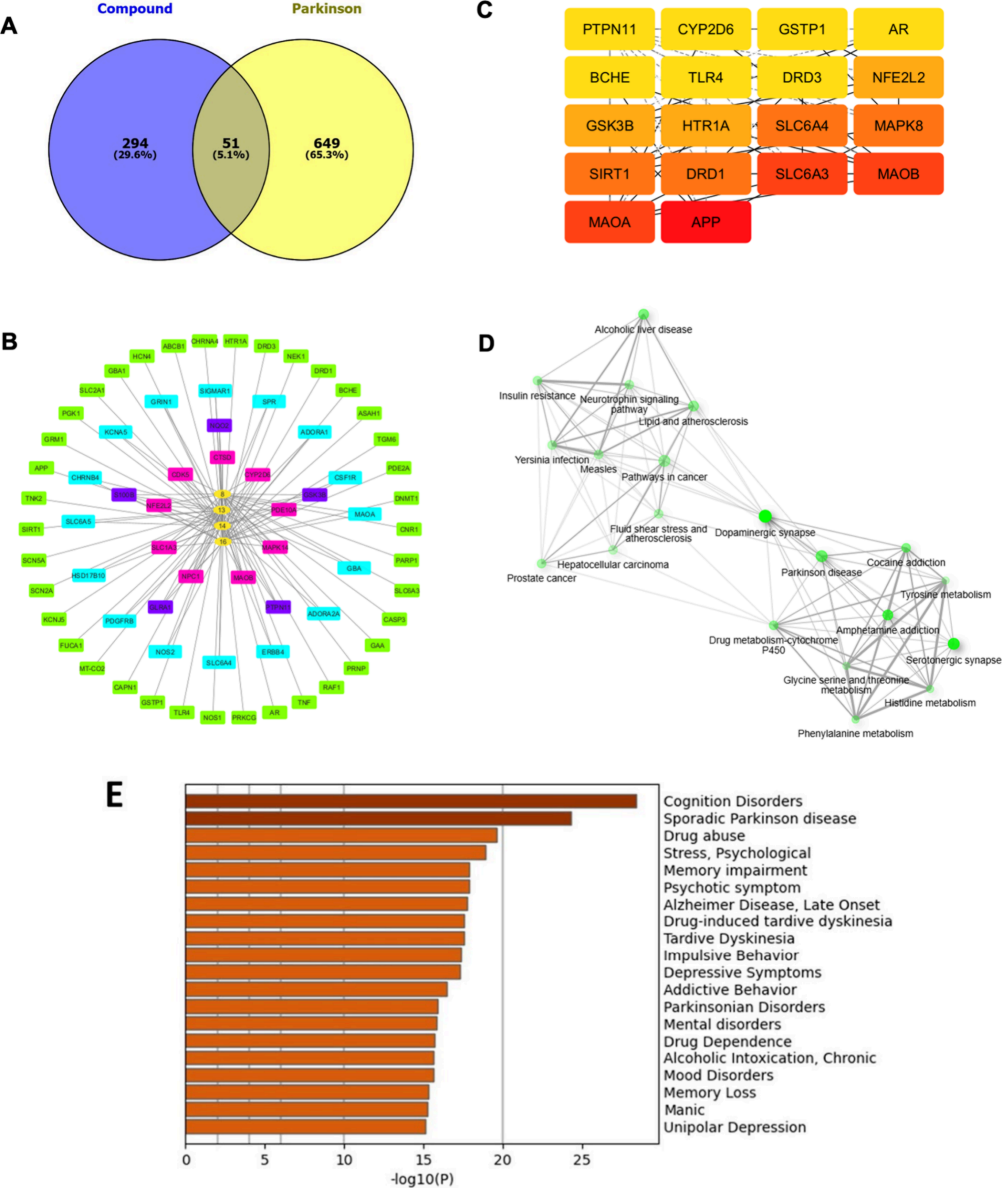
*In silico* network pharmacology of acetamidosulfonamides and PD. (A) Venn diagram illustrating the intersection between the target proteins of the compounds **8**, **13**, **14**, and **16** and PD. The constructions of (B) CTD and (C) PPI networks using Cytoscape. The top 20 network pathways (D) illustrating and (E) scoring by ShinyGO and GO analyses.

**Table 1:** Analysis of the Top 18 Target-Associated Acetamidosulfonamides and PD^a^

**no.**	**protein**	**gene**	**mechanism**	**DC**	**BC**	**CC**	**network**	**ref**
1	amyloid-beta precursor protein	APP	cell surface receptor, regulator of synapse formation, neural plasticity, antimicrobial activity, and iron export	7	686.33	0.32	0	^29^
2	monoamine oxidase A	MAOA	oxidative deamination of amine neurotransmitters (i.e., serotonin, norepinephrine, and epinephrine) and dietary amines	6	282.80	0.28	3.90	^30^
3	monoamine oxidase B	MAOB	oxidative deamination of amine neurotransmitters (i.e., phenethylamine and benzylamine) and dietary amines	6	282.80	0.28	3.90	^30^
4	sodium-dependent dopamine transporter	SLC6A3	mediation of sodium- and chloride-dependent transport of dopamine and norepinephrine	6	268.00	0.25	2.22	^31^
5	D(1A) dopamine receptor	DRD1	dopamine receptor mediated by G proteins activates adenylyl cyclase	5	332.80	0.22	1.50	^3^
6	NAD-dependent protein deacetylase sirtuin 1	SIRT1	cellular functions (i.e., cell cycle, DNA damage, metabolism, apoptosis, and autophagy)	5	114.00	0.24	2.08	^22^
7	mitogen-activated protein kinase 8	MAPK8	cell proliferation, differentiation, migration, transformation, and programmed cell death	5	431.67	0.30	1.42	^32^
8	sodium-dependent serotonin transporter	SLC6A4	regulation of serotonergic signaling, neurotransmitter transmembrane transporter activity, and nitric oxide synthase binding activity	5	196.00	0.25	2.42	^31^
9	5-hydroxytryptamine receptor 1A	HTR1A	G protein-coupled serotonin receptor activity.	4	0.80	0.24	3.33	^33^
10	glycogen synthase kinase-3 beta	GSK3B	involvement in energy metabolism, neuronal cell development, and body pattern formation	4	134.67	0.27	1.50	^34^
11	nuclear factor erythroid 2-related factor 2	NFE2L2	regulation of metabolism, inflammation, autophagy, proteostasis, mitochondrial physiology, and immune responses	4	31.00	0.26	2.17	^35^
12	D(3) dopamine receptor	DRD3	regulation of dopamine release and modulation of neuronal excitability	3	0.80	0.21	2.00	^3^
13	Toll-like receptor 4	TLR4	activator of the innate immune response both pathogen- and damage-associated molecular patterns (PAMPs and DAMPs)	3	282.00	0.22	0	^36^
14	butyrylcholinesterase	BCHE	inactivation of the neurotransmitter acetylcholine	3	439.67	0.30	1.00	^37^
15	androgen receptor	AR	negative regulation of androgen receptor signaling and androgen-induced cell proliferation	3	72.00	0.23	1.00	^38^
16	glutathione *S*-transferase P	GSTP1	involvement in the formation of glutathione conjugates of both prostaglandin A2 and J2	3	176.33	0.28	1.00	^39^
17	cytochrome P450 2D6	CYP2D6	metabolism of fatty acids, steroids, and retinoids	3	170.33	0.27	1.00	^40^
18	tyrosine-protein phosphatase nonreceptor type 11	PTPN11	regulation of cell signaling pathways (i.e., cell proliferation, differentiation, migration, and survival)	3	142.00	0.16	0	^41^

a DC = degree centrality, BC = betweenness centrality, and CC = closeness centrality.

**Table 2:** Enrichment Analysis of Acetamidosulfonamides

**GO**	**pathway**	**count**	**%**	Log 10(*P*)	Log 10(*q*)
C0009241	cognition disorders	18	90	–28	–24
C4511452	sporadic PD	13	65	–24	–20
C0013146	drug abuse	13	65	–20	–16
C0038443	psychological stress	11	55	–19	–15
C0233794	memory impairment	14	70	–18	–14
C0871189	psychotic symptoms	9	45	–18	–14
C0494463	late-onset AD	12	60	–18	–14
C0686347	Tardive dyskinesia	9	45	–18	–14
C3714760	drug-induced tardive dyskinesia	9	45	–18	–14
C0021125	impulsive behavior	11	55	–17	–14
C0086132	depressive symptoms	12	60	–17	–14
C0085281	addictive behavior	11	55	–16	–13
C0242422	Parkinsonian disorders	11	55	–16	–12
C0004936	mental disorders	13	65	–16	–12
C1510472	drug dependence	10	50	–16	–12
C0001973	chronic alcoholic intoxication	12	60	–16	–12
C0525045	mood disorders	12	60	–16	–12
C0751295	memory loss	9	45	–15	–12
C0338831	manic	9	45	–15	–12
C0041696	unipolar depression	12	60	–15	–12

### 3.6 Acetamidosulfonamides Possess Drug-Likeness Properties

Physicochemical and pharmacokinetic features of the four compounds (**8**, **13**, **14**, and **16**) were computationally calculated to assess their drug-likeness and bioavailability capabilities. The results showed that all compounds are drug-like, obeying the Lipinski rule of five and displaying molecular weight (MW) less than 500 g/mol, partition coefficient (Log *P*) less than 5, and hydrogen bond donors (HBDs) and acceptors (HBAs) less than 5 and 10, respectively^42^ (Table 3). Additionally, the calculated properties were also in line with other rules (i.e., Ghose,^43^ Veber,^44^ Egan,^45^ and Muegge^46^), suggesting that these compounds are preferable for possible drug development.

**Table 3:** Physicochemical Properties of Acetamidosulfonamides

**compound**	8	**13**	**14**	**16**
MW (g/mol)	284.33	318.34	378.44	313.42
partition coefficient (Log *P*)	0.61–1.76	2.17–3.71	2.18–3.73	1.27–2.81
H-bond acceptor (HBA)	5	4	6	5
H-bond donor (HBD)	1	2	2	2
molar refractivity (MR)	74.05	85.94	98.93	83.58
rotatable bond (RB)	4	7	9	9
topological polar surface area (TPSA)	84.09–111.96	83.65–129.97	102.11–152.93	86.89–120.13
Lipinski’s rule	yes	yes	yes	yes
Ghose’s rule	yes	yes	yes	yes
Veber’s rule	yes	yes	yes	yes
Egan’s rule	yes	yes	yes	yes
Muegge’s rule	yes	yes	yes	yes

*In silico* pharmacokinetic and toxic models predicted that the four investigated compounds are water/lipid-soluble, highly absorbed in the gastrointestinal (GI) tract, low skin permeable, and vary in Caco2 permeabilities, indicating their potentials for oral administration. All compounds may adequately pass across the blood–brain barrier (BBB) and central nervous system (CNS), except for the high BBB permeability of compound **13**, suggesting their abilities to reach the target site of action for PD treatment. Compound **14** was shown to be an inhibitor of all investigated CYP450 enzymes, except for CYP1A2, which indicated its potential for drug–drug interactions. In contrast, the rest of the compounds (**8**, **13**, and **16**) were predicted as non-inhibitors of most of the investigated CYP450s, suggesting their lesser drug–drug interaction potentials. All compounds were predicted as non-substrates of renal organic cation transporters 2 (OCT2) with a total clearance range of 0.629–0.749 Log mL/min/kg. The compounds displayed predicted median lethal dose (LD_50_) ranging from 87 to 3000 mg/kg body weight. There were no predicted toxicities (i.e., hepatotoxicity, carcinogenicity, immunotoxicity, mutagenicity, and cytotoxicity), which is consistent with the *in vitro* neurotoxicity by MTT assay, LDH, and apoptotic profiles (Table 4). Among others, compound **16** was shown to be the most preferable drug-like compound with less potential drug–drug interactions and toxicities (as predicted to be a non-inhibitor of CYP450s and a non-substrate of OCT2, as well as its highest LD_50_).

**Table 4:** Pharmacokinetic Properties of Acetamidosulfonamides

**compound**	**8**	**13**	**14**	**16**
Absorption				
lipophilicity (iLOGP)	soluble	soluble	soluble	soluble
water solubility	soluble	soluble	soluble	soluble
GI absorption	high	high	high	high
Caco2 permeability (Log Papp)	low	adequate	high	high
skin permeability (Log Kp)	low	low	low	low
Distribution				
BBB permeability (Log BB)	adequate	high	adequate	adequate
CNS permeability (Log PS)	adequate	adequate	adequate	adequate
human VDss (Log L/kg)	–0.384	–0.09	–0.137	0.607
Metabolism				
CYP1A2 inhibitor	no	no	no	no
CYP2C19 inhibitor	no	no	yes	no
CYP2C9 inhibitor	no	no	yes	no
CYP2D6 inhibitor	no	yes	yes	no
CYP3A4 inhibitor	no	no	yes	no
Elimination				
total clearance (Log mL/min/kg)	0.629	0.749	0.686	0.646
renal OCT2 substrate	No	no	no	no
Toxicity				
predicted LD_50_ (mg/kg)	580	87	1000	3000
predicted toxicity class	4	3	4	5
hepatotoxicity	inactive	inactive	inactive	inactive
carcinogenicity	inactive	inactive	inactive	inactive
immunotoxicity	inactive	inactive	inactive	inactive
mutagenicity	inactive	inactive	inactive	inactive
cytotoxicity	inactive	inactive	inactive	inactive

## 4 Discussion

Current pharmaceutical industries have been directed to search for modern approaches to rapidly screen potent candidates and efficiently validate for drug discovery and modification, including preclinical and clinical studies, before launching to the pharmaceutical markets. Computational strategies have been implemented as a means to overcome pharmaceutical obstacles in drug discovery and development by minimizing processing length, labor, and cost, ultimately leading to a high success rate for clinical use.^47^ For this reason, the present study utilized experimental biological assessments in conjunction with computational tools, offering a comprehensive elucidation of anti-PD mechanisms of the compounds against 6-OHDA-triggered neurotoxicity in human neuronal SH-SY5Y cells. 6-OHDA, a neurotoxic analog of the oxidizable DA taken up by the DA transporter, selectively damages the catecholaminergic neurons, allowing Parkinsonian mimicking both *in vitro* and *in vivo* experiments to study the neuropathological hallmarks of PD, particularly loss of DA neurons in the SNpc, α-syn misfolding, Lewy body aggregation, neuroinflammation, and mitochondria-mediated apoptosis.^8–10,48^ Although the investigated compounds demonstrated their antioxidant activities,^6^ the complete mechanistic PD understanding remains limited in alleviating 6-OHDA *via* modulation of the SIRT1-AChE signaling axis.

Based on the *in vitro* experiments, no cytotoxicity of the synthetic acetamidosulfonamides (**1**–**16**) was observed at various concentrations ranging from 0.1 to 100 μM, compared to the untreated SH-SY5Y cells. In addition, these compounds were shown to be non-cytotoxic to the normal lung MRC-5 cells. Among the investigated compounds, pretreatment with ethylamino moieties (R), i.e., **13**, **14**, and **16**, displayed the highest efficacy in the sublethal window of 6-OHDA-toxicated cells, followed by cyclic amino (**8**). The results were further supported by the minimal numerical and morphological changes of the neuronal cells pretreated with the compounds of interest, compared to the 6-OHDA exposure. Notably, the sulfonamides (i.e., **8**, **13**, **14**, and **16**) modulated a dynamic interplay of oxidative stress and programmed cell death, including ROS generation, MMP activity, LDH leakage, and cell apoptosis, resulting in the enhancement of neuronal health, in comparison to the well-established RSV antioxidant.

Traditionally, symptomatic medications of NDs, especially AD, mainly rely on AChE inhibitors to break down the major ACh neurotransmitter in the cholinergic system, leading to ACh accumulation at synapses.^4^ Several lines of research have reported that AChE activity is significantly decreased in patients with early PD dementia and cognitive impairment, owing to the above-mentioned cholinergic degeneration and contributing to defects in motor function, as well as further triggering several underlying biological mechanisms.^49^ The reduction of AChE activity and induction of ROS by 6-OHDA exposure in this finding aligned with the previous research, implying the potential neurotoxicity of 6-OHDA on the cholinergic system. Importantly, the synthetic acetamidosulfonamides (**8**, **13**, **14**, and **16**) attempted to physically maintain the cellular function by increasing the AChE activity similar to that of the RSV. *In silico* molecular docking also supported the *in vitro* AChE enzymatic observations. All selected compounds simultaneously bind the dumbbell-shaped AChE, both PAS and CAS domains, with the same binding site as donepezil, sharing the common key interacting amino acid residues (i.e., Ser293, Phe295, Phe338, and Val294).^50^ The presence of benzene rings and sulfonyl sulfur groups in the molecules **14** and **13** was noted to be essential for anchoring Phe297 and Phe338 in the mid-Gorge stabilized by π-based interactions, whereas carbonyl or sulfonyl oxygens played key roles in hydrogen bonding interactions extended toward Tyr124 in the PAS. These inhibitors provide a synergistic dual-action therapeutic strategy by enhancing the AChE inhibition and blocking protein aggregation, which is considered essential for treating NDs.^51^ However, the binding free energy of these compounds was less preferable than that of donepezil. Further investigation into the possible inhibitory effect on BChE of these acetamidosulfonamides is recommended according to the previously reported potent dual inhibitory effects on both AChE and BChE by the hydrazone-derived 4-acetamidobenzenesulfonyl group.^52^

Regarding the SIRT1-related neuroprotection by acetamidosulfonamides in the Parkinsonian condition, both *in vitro* SIRT1 activation and localized observations, as well as *in silico* SIRT1 binding capability, were extensively performed. The SIRT1 enzymatic results revealed that the 6-OHDA treatment markedly decreased the SIRT1 activity and fluorescent signal in human neuronal cells, whereas the four derivatives **8**, **13**, **14**, and **16** act as SIRT1 activators like the reference RSV by significantly reversing the neuronal damage. In addition, the potent role of SIRT1 activators was further confirmed by the blockage of SIRT1 using a SIRT1 inhibitor, sirtinol, for 1 h prior to the cotreatment of acetamidosulfonamides and 6-OHDA. Sirtinol itself did not affect the total apoptosis, while inhibition of the SIRT1 enzyme by sirtinol was observed, compared to the untreated cells. These were consistent with the previous studies in MPP^+^-treated SH-SY5Y cells and oxygen-glucose deprivation/reoxygenation-induced N9 microglia, referring to the oxidative stress system upon SIRT1 suppression.^53^ Furthermore, the inhibitory effect of sirtinol was remarkably hindered by acetamidosulfonamides, resulting in the reduction of apoptotic rates under 6-OHDA conditions, as represented in the apoptotic profiles and SIRT1 enzymatic assay, indicating the modulation of SIRT1 and its downstream pathway by the neuroprotective effect of acetamidosulfonamides against 6-OHDA-induced Parkinsonian conditions. In agreement with these findings, the strong consistency between the SIRT1-targeted molecular docking and previously reported pharmacological properties of sulfonamide-based pharmacophores^8–10^ supports the neuroprotective potential of these acetamidosulfonamides (**8**, **13**, **14**, and **16**) as SIRT1 activators comparable to that of the RSV molecule. The preferable binding with SIRT1 could be due to the presence of benzene rings that mimic those terminal rings of the RSV for π-interactions with Leu202, Pro211, and Ile223. Similar to the binding against AChE, the sulfonyl or carbonyl oxygen atom was noted for crucial conventional hydrogen bonding with Asn226. Additionally, the superior binding behavior of compound **14** among others could be due to the substituted side chain containing two methoxy (−OCH_3_) groups on its terminal benzene ring playing a role in an additional hydrogen bond formation with Thr209. The findings were also supported by previously reported literature investigating the effect of antioxidant and SIRT1 activation of RSV on regulating the downstream pathway of neuronal longevity.^54^

Apart from the cholinergic system and SIRT1-regulated pathways, an integrative approach combining network pharmacology and bioinformatics was employed to reveal the possible target-related biological processes and cooperate in the PD pathology. There are 51 overlapping targets from 700 targets of human PD and 345 acetamidosulfonamides-associated targets, which may accompany function in regulating pathways of PD neuroprotection. Through the above-mentioned CTD and PPI analyses of 51 potential targets, APP, MAOA, MAOB, SLC6A3, and DRD1 are the top five proteins predominantly associated with the DA signaling pathway commonly related to several conditions, i.e., schizophrenia,^55^ PD,^56^ depression,^57^ and alcohol dependence.^58^ These underlying diseases are in agreement with the current work of pathway enrichment analysis by ShinyGO pathway and GO enrichment analyses. Particularly, SIRT1 and BChE emerge as the top 18 key targets implicated in acetamidosulfonamides-mediated PD protection. Collectively, these relevant data strongly support the idea that the synthetic acetamidosulfonamides (**8**, **13**, **14**, and **16**) activate the SIRT1-DA signaling pathway, which regulates multiple cellular function-mediated PD, including cell survival,^38,41^ apoptosis,^32^ oxidative stress,^30,39^ inflammation,^35,36^ mitochondrial function,^34,40^ and the DA system.^3,31,33,37^ These findings underscore the potential major role of SIRT1-DA signaling in the therapeutic effects of acetamidosulfonamides on PD and can be extended to the relevant area of therapeutics.

Potential for future development of the synthetic compounds **8**, **13**, **14**, and **16** was also highlighted by their predicted pharmacokinetic features with no violations for all drug-likeness rules^42–46^ as well as high probability of GI absorption, adequate penetration of the BBB and CNS. The results suggested the possibility of developing these compounds as oral drugs with less severe toxicities. Our findings are consistent with the previous studies reporting the preferable drug-likeness of some acetamidosulfonamides-based anti-inflammatory agents (i.e., acetamide–sulfonamide-conjugated nonsteroidal anti-inflammatory drugs (NSAIDs)^59^ and *N*-substituted-2-((2-oxo-2-((4-sulfamoylphenyl)­amino)­ethyl)­thio) acetamide derivatives^60^). Overall, these preclinical findings suggest that these synthetic acetamidosulfonamides might be promising candidates for the treatment and prevention of NDs. The valuable insights into the association between PD pathogenesis and cholinergic dysfunction are provided in Figure 8. Although employing *in silico* approaches is capable of boosting the drug discovery and development processes, there still remain several drawbacks. Therefore, the incorporation of an artificial intelligence (AI)-driven drug discovery platform, in-depth experimental research, and comprehensive validation *in vivo* should be warranted.

**Figure 8:**
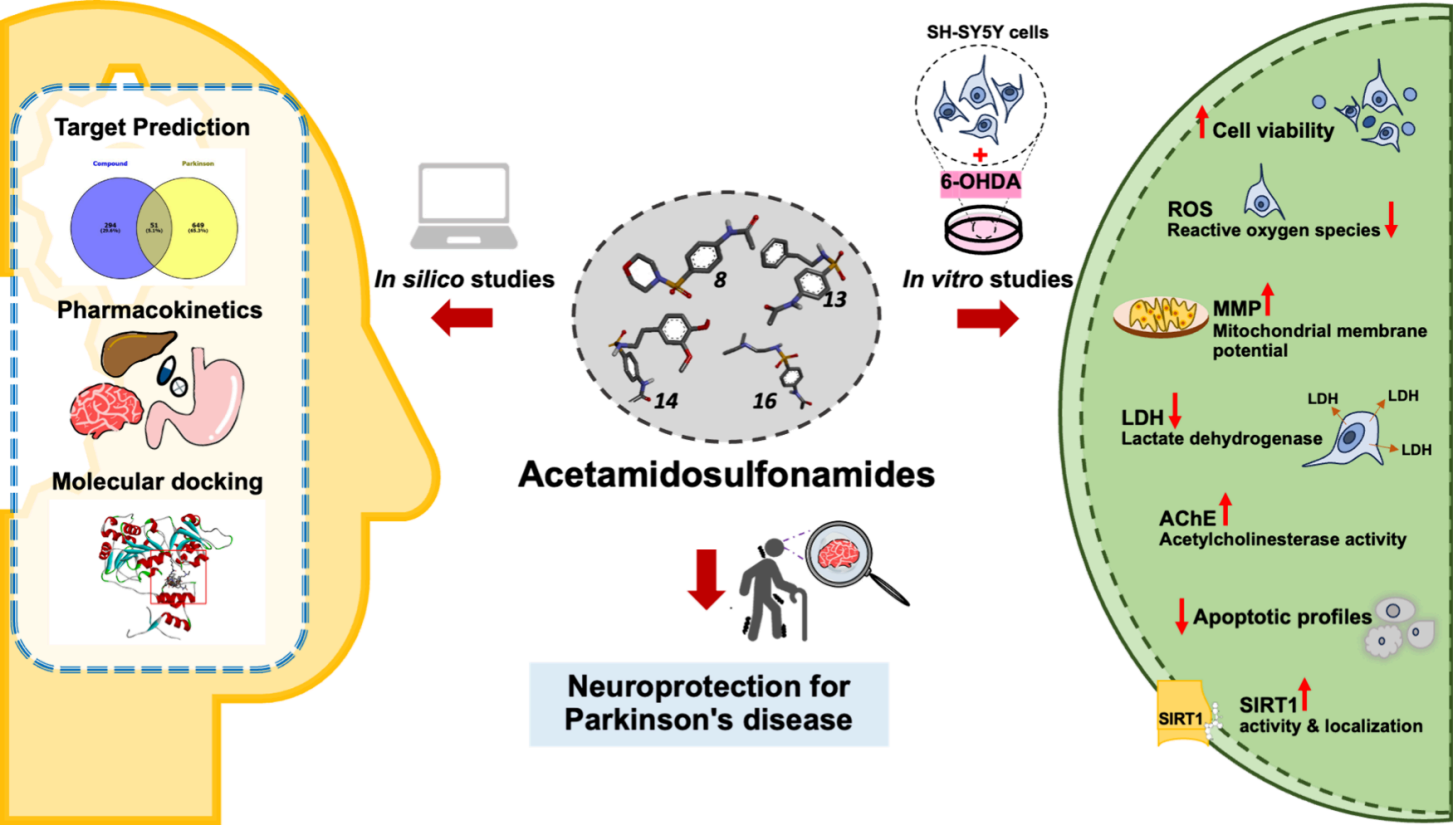
Proposed underlying mechanisms of acetamidosulfonamides against 6-OHDA-induced Parkinsonian model in SH-SY5Y cells *via* enhancing antioxidant, maintaining different cellular components, balancing AChE activity, and regulating SIRT1 protein.

## 5 Conclusions

The current study, using a variety of biological and *in silico* tools, including an *in vitro* SH-SY5Y model, molecular docking, ADMET prediction, network pharmacology, and target enrichment analysis, could pave the way for future investigations on the precise molecular mechanistic pathways driven by the four synthesized acetamidosulfonamides (**8**, **13**, **14**, and **16**). The results showed that the tested compounds have neuroprotective properties against 6-OHDA-induced neurodegeneration in SH-SY5Y cells by activating the SIRT1-mediated AChE signaling pathway. Several key targets (i.e., APP, SLC6A4, MAOB, MAOA, and DRD1) are directly involved in regulating the neurotransmitters and oxidative stress. The molecular docking study revealed that compound **14** exhibited the most favorable binding behavior against both SIRT1 and AChE targets, which might be due to the presence of a terminal benzene ring substituted with a di-OCH_3_ group. Sulfonyl oxygen was also noted as a key moiety for binding to the CAS and PAS domains of AChE via π–sulfur and π–cation interactions. Furthermore, these synthetic sulfonamides possess drug-likeness with no violation. These conclusions may provide promising anti-PD candidates of the acetamidosulfonamides for the prevention and treatment of the devastating PD progression, as well as a novel scientific basis for the development and utilization of the core pharmacophores. More target-specific molecular mechanisms are needed to uncover the probable underlying PD, which will be transferred from preclinical data to clinical settings.

## Supplementary Material



## Notes

Immunofluorescent imaging, molecular docking, network pharmacology, and cytotoxic activities of acetamidosulfonamides (**1**–**16**) (PDF)
