# Supplementary material for: Molecular Mechanisms and Network Pharmacology Revealing Therapeutic Potential of Acetamidosulfonamides against Parkinsonian Model
Source: ACS Omega. 2026 May 21;11(21):31459–76. doi: 10.1021/acsomega.6c01936 (PMC13234791; doi:10.1021/acsomega.6c01936)
Supplement: Supplementary file 1 [file ao6c01936_si_001.pdf]

Supplementary data

Molecular Mechanisms and Network Pharmacology Revealing Therapeutic Potential of Acetamidosulfonamides against Parkinsonian Model

Waralee Ruankham[1], Veda Prachayasittikul[2], Ratchanok Pingaew[3], Tanawut Tantimongcolwat[2], Apilak Worachartcheewan[4], Kanyarat Chompon[2], Somsak Ruchirawat[5,6,7], Virapong Prachayasittikul[8], Supaluk Prachayasittikul[2], Kamonrat Phopin[2,8,*]

[1]Department of Clinical Chemistry, Faculty of Medical Technology, Mahidol University, Bangkok, 10700, Thailand
[2]Center for Research Innovation and Biomedical Informatics, Faculty of Medical Technology, Mahidol University, Bangkok, 10700, Thailand
[3]Department of Chemistry, Faculty of Science, Srinakharinwirot University, Bangkok, 10110, Thailand
[4]Department of Community Medical Technology, Faculty of Medical Technology, Mahidol University, Bangkok, 10700, Thailand
[5]Laboratory of Medicinal Chemistry, Chulabhorn Research Institute, Bangkok, 10210, Thailand
[6]Program in Chemical Sciences, Chulabhorn Graduate Institute, Bangkok, 10210, Thailand
[7]Center of Excellence on Environmental Health and Toxicology (EHT), Commission on Higher Education, Ministry of Education, Bangkok, 10400, Thailand
[8]Department of Clinical Microbiology and Applied Technology, Faculty of Medical Technology, Mahidol University, Bangkok, 10700, Thailand

*Corresponding author: Kamonrat Phopin
E-mail: kamonrat.php@mahidol.ac.th

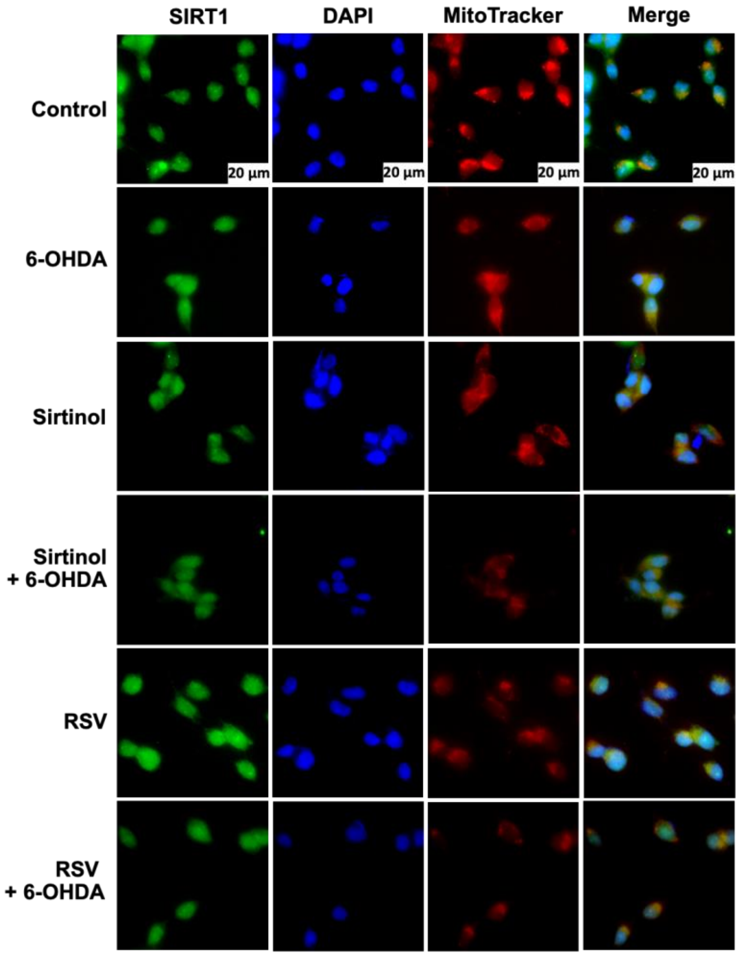

Figure S1. Localization of SIRT1 in sirtinol or RSV-pretreated SH-SY5Y cells at 20× magnification.

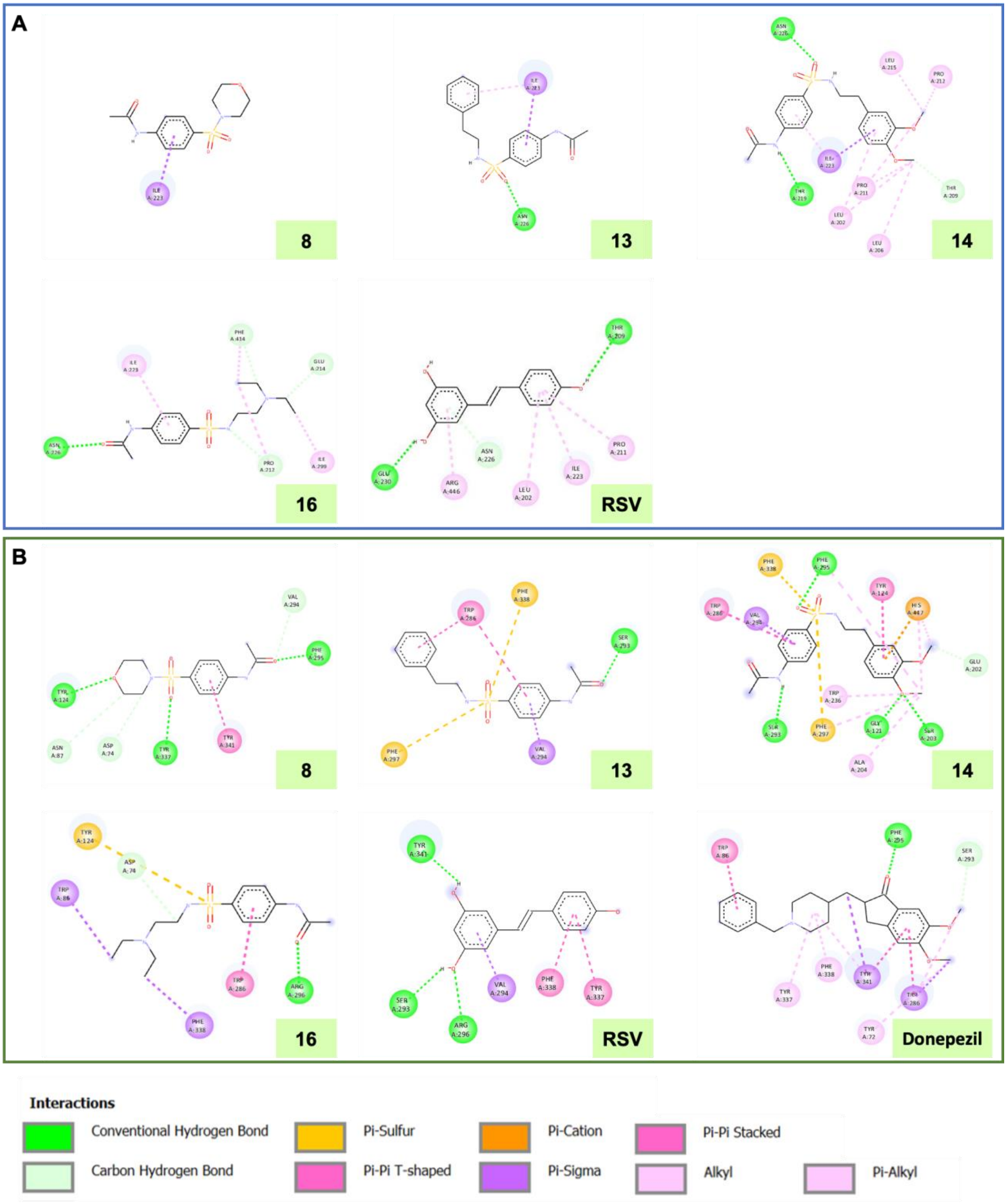

Figure S2. Molecular docking of acetamidosulfonamides in the active binding site of (A) SIRT1 and (B) AChE. The interacting amino acid residues are depicted as colored circles based on the hydrophilic and hydrophobic types of interaction.

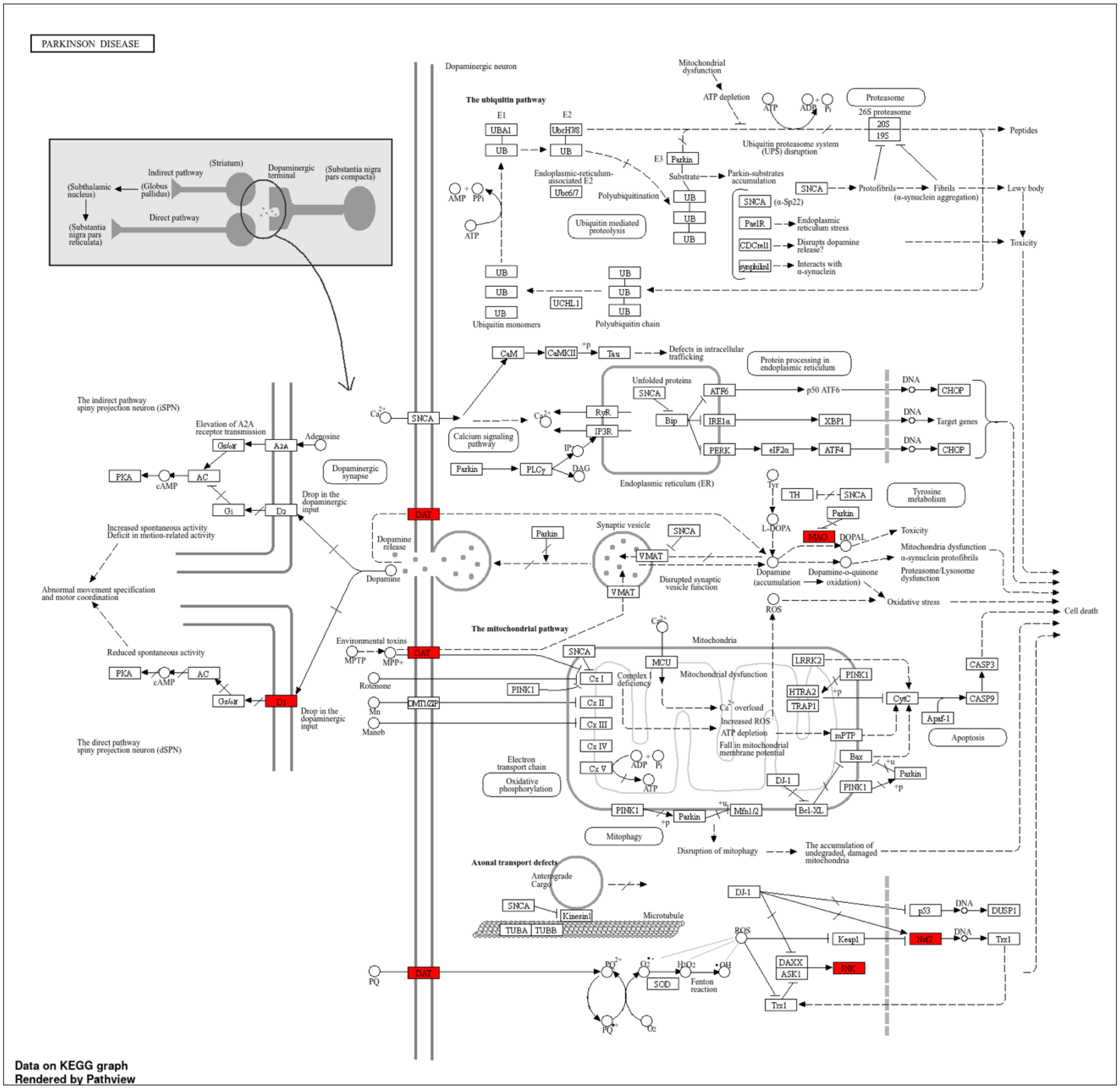

Figure S3. Illustration of PD pathway driven by *in silico* network pharmacology of acetamidosulfonamides.

Table S1. Cytotoxic activities of acetamidosulfonamide (**1-16**) against human lung fibroblast (MRC-5) cells.

Group	Compound	MRC-5 cells (IC_{50}, µg/mL)[a]	Cytotoxic activity[b]
Methylamino	1	4.16	Non-cytotoxic
	2	0.00	Non-cytotoxic
	3	0.00	Non-cytotoxic
	4	0.00	Non-cytotoxic
Cyclic amino	5	0.40	Non-cytotoxic
	6	0.00	Non-cytotoxic
	7	10.10	Non-cytotoxic
	8	0.00	Non-cytotoxic
	9	1.73	Non-cytotoxic
Ring-substituted amino	10	2.63	Non-cytotoxic
	11	0.00	Non-cytotoxic
	12	2.60	Non-cytotoxic
Ethylamino	13	12.96	Non-cytotoxic
	14	6.63	Non-cytotoxic
	15	0.06	Non-cytotoxic
	16	0.00	Non-cytotoxic
Reference	Doxorubicin hydrochloride[c]	1.31 ± 0.13	Cytotoxic

[a]IC_{50} is the concentration of compound required to produce 50 % of inhibitory effect.

[b]Non-cytotoxic effect is denoted when inhibitory effect greater than 50 % at 50 µg/mL.

[c]Doxorubicin hydrochloride was used as a reference drug.
